# Community Composition and Abundance of Bacterial, Archaeal and Nitrifying Populations in Savanna Soils on Contrasting Bedrock Material in Kruger National Park, South Africa

**DOI:** 10.3389/fmicb.2016.01638

**Published:** 2016-10-19

**Authors:** Saskia Rughöft, Martina Herrmann, Cassandre S. Lazar, Simone Cesarz, Shaun R. Levick, Susan E. Trumbore, Kirsten Küsel

**Affiliations:** ^1^Chair of Aquatic Geomicrobiology, Institute of Ecology, Friedrich Schiller University JenaJena, Germany; ^2^German Centre for Integrative Biodiversity Research (iDiv) Halle-Jena-LeipzigLeipzig, Germany; ^3^Institute of Biology, Leipzig UniversityLeipzig, Germany; ^4^Biogeochemical Processes, Max Planck Institute for BiogeochemistryJena, Germany

**Keywords:** savanna soils, ammonia-oxidizing bacteria, ammonia-oxidizing archaea, amoA, granitic bedrock, basaltic bedrock

## Abstract

Savannas cover at least 13% of the global terrestrial surface and are often nutrient limited, especially by nitrogen. To gain a better understanding of their microbial diversity and the microbial nitrogen cycling in savanna soils, soil samples were collected along a granitic and a basaltic catena in Kruger National Park (South Africa) to characterize their bacterial and archaeal composition and the genetic potential for nitrification. Although the basaltic soils were on average 5 times more nutrient rich than the granitic soils, all investigated savanna soil samples showed typically low nutrient availabilities, i.e., up to 38 times lower soil N or C contents than temperate grasslands. Illumina MiSeq amplicon sequencing revealed a unique soil bacterial community dominated by *Actinobacteria* (20–66%), *Chloroflexi* (9–29%), and *Firmicutes* (7–42%) and an increase in the relative abundance of *Actinobacteria* with increasing soil nutrient content. The archaeal community reached up to 14% of the total soil microbial community and was dominated by the thaumarchaeal Soil Crenarchaeotic Group (43–99.8%), with a high fraction of sequences related to the ammonia-oxidizing genus *Nitrosopshaera* sp. Quantitative PCR targeting *amoA* genes encoding the alpha subunit of ammonia monooxygenase also revealed a high genetic potential for ammonia oxidation dominated by *archaea* (~5 × 10^7^ archaeal *amoA* gene copies g^−1^ soil vs. mostly < 7 × 10^4^ bacterial *amoA* gene copies g^−1^ soil). Abundances of archaeal 16S rRNA and *amoA* genes were positively correlated with soil nitrate, N and C contents. *Nitrospira* sp. was detected as the most abundant group of nitrite oxidizing bacteria. The specific geochemical conditions and particle transport dynamics at the granitic catena were found to affect soil microbial communities through clay and nutrient relocation along the hill slope, causing a shift to different, less diverse bacterial and archaeal communities at the footslope. Overall, our results suggest a strong effect of the savanna soils' nutrient scarcity on all microbial communities, resulting in a distinct community structure that differs markedly from nutrient-rich, temperate grasslands, along with a high relevance of archaeal ammonia oxidation in savanna soils.

## Introduction

As the most common (sub-) tropic vegetation type, savannas cover over 13% of the global terrestrial surface and host 20% of the current human population (Bustamante et al., 2006). Savanna ecosystems are generally defined by a continuous grass/herbaceous layer which coexists with trees and shrubs of varying density, often accompanied by a strong wet/dry seasonality and differing fire regimes (Scholes and Archer, 1997). Due to long dry periods, highly weathered or nutrient-poor tropical soils and the impact of periodical wildfires, water and nutrient availability generally determine the structure and function of savanna ecosystems, with nitrogen (N) availability being particularly limiting in dry savannas (Scholes and Archer, 1997; Bustamante et al., 2006; Cech et al., 2008). Nutrient cycling in terrestrial systems largely depends on soil microbial communities, which perform nutrient transformations and drive biogeochemical cycles on local and global scales (Bardgett et al., 2005; Falkowski et al., 2008; van der Heijden et al., 2008; Schimel and Schaeffer, 2012). Several abiotic and biotic factors like soil mineralogy, pH, water content, climate, seasons, vegetation or herbivory can determine soil microbial community structure and composition (Myers et al., 2001; Bardgett et al., 2005; Fierer and Jackson, 2006), which in turn have been linked to changes in microbially mediated processes and the corresponding ecosystem functions (Waldrop et al., 2000; Braker and Conrad, 2011; Bier et al., 2015).

Concordantly, most of the biogeochemical redox processes forming the terrestrial N cycle are catalyzed directly by soil-dwelling microorganisms, e.g., nitrifiers and denitrifiers (Falkowski et al., 2008; van der Heijden et al., 2008; Ollivier et al., 2011). Ammonia oxidation as the first, rate-limiting step of nitrification is performed by members of the *Beta*- and *Gammaproteobacteria* (AOB) and *Thaumarchaeota* (AOA) (Koops et al., 2003; Könneke et al., 2005). The second step of nitrification is performed by various *Proteobacteria* and members of *Nitrospirae*, which together form the polyphyletic group of nitrite-oxidizing bacteria (NOB) (Abeliovich, 2006). Recently, it was also discovered that some members of the *Nitrospira* genus can perform both steps, i.e., a complete nitrification (comammox), but their ecological relevance remains unclear at this point (Daims et al., 2015; van Kessel et al., 2015). The *amoA* gene encoding the catalytic alpha subunit of ammonia monooxygenases of AOB or AOA has been used as a molecular marker to target AOA and AOB in a vast number of environmental surveys (Rotthauwe et al., 1997; Francis et al., 2005), recently complemented by the *nxrB* gene encoding nitrite oxidoreductase of nitrite oxidizers of the genus *Nitrospira* (Pester et al., 2014).

The majority of available data on soil microbial communities has been obtained in temperate soil environments where *Proteobacteria, Acidobacteria*, and *Actinobacteria* typically dominate the bacterial communities while mainly *Thaumarchaeota* (formerly classified as mesophilic *Crenarchaeota*) and a few *Euryarchaeota* make up the archaeal communities (Janssen, 2006; Timonen and Bomberg, 2009; Will et al., 2010; Bates et al., 2011; Nacke et al., 2011). However, savanna and (semi-)arid shrub land soil microbial community compositions and functions, especially related to N cycling, have only rarely been addressed (Araujo et al., 2012; Catão et al., 2013; Rampelotto et al., 2013; Huber, 2015; Mueller et al., 2015; Ronca et al., 2015). Here, studies regarding central factors that shape microbial communities in savanna soils involved in N cycling have remained inconclusive (Rachid et al., 2012; Braker et al., 2015).

This study aimed to elucidate microbial diversity and nitrifying soil microbial communities in subtropical savanna soils by using the Kruger National Park (KNP), South Africa, as a model environment. KNP is located in the northeastern part of South Africa and encompasses almost 2 million ha of subtropical deciduous savanna landscapes inhabited by intact herbivore and carnivore populations, ensuring integrity of most ecosystem processes. Geologically, the western half of KNP lies on Kaapval granite and metagranites while basalts (Karoo volcanics) underlie the eastern half (Venter and Gertenbach, 1986; Venter et al., 2003; Coetsee et al., 2012; Chadwick et al., 2013). Recently, several “research supersites” were established in order to facilitate research on representative non-manipulated savanna landscapes in the park (Smit et al., 2013). Two soil catenas on granitic and basaltic bedrock, respectively, were chosen as study sites within the KNP's research supersite network. Catenas are hydrologically linked hillslope soils with dynamic solute, particle and colloid transport processes, leading to soil differentiation along their slopes, which was more pronounced at the granitic study site than at the less well-differentiated basaltic catena (Milne, 1935; Khomo et al., 2011). Together with the savanna's typical wet/dry seasonality, these processes additionally lead to seasonal seep zones at the granitic site.

We expected a strong impact of the savanna soils' nutrient-poor status on soil microbial community structure and expected these interactions to be further modulated by bedrock material as well as by the spatial heterogeneity resulting from the described catena dynamics. Given the crucial role of nitrogen in nutrient cycling of these nutrient-poor soils, we were specifically interested in how these factors would shape microbial communities involved in nitrification. Consequently, the objectives of this study were (i) to identify to what extent microbial community structure of the KNP savanna soils differs from those usually found in temperate grassland soils, (ii) to assess the impact of the sites' contrasting bedrock material and resulting soil geochemistry and catena dynamics on the soil microbial communities and (iii) to evaluate the genetic potential for nitrification by targeting *amoA* and *nxrB* genes as molecular markers.

## Materials and methods

### Study sites and sampling procedure

**Figure 1:**
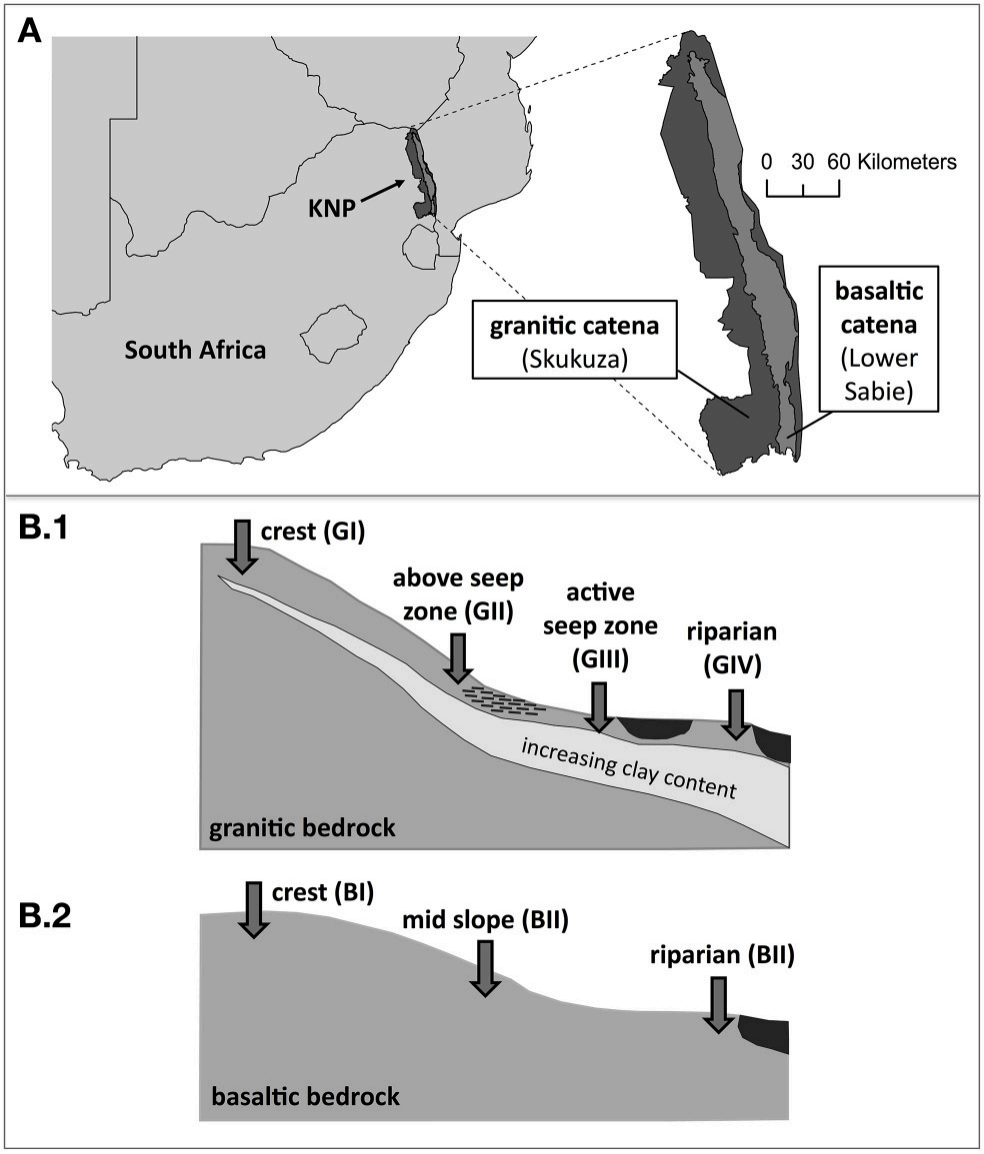
**Sampling sites. (A)** Schematic map of southern Africa with Kruger National Park (KNP), a simplified overview of the park's geology and locations of both sampled supersites near Skukuza and Lower Sabie, respectively. **(B)** Schematic overview of the sampling points in 5 cm depths along the two catenas on geologically different bedrock. **(B1)** Sampling locations at granitic catena: crest (GI), above seep zone (GII), active seep zone (GIII), riparian zone (GIV). **(B2)** Sampling locations at basaltic catena: crest (BI), mid slope (BII), riparian zone (BIII).

The samples investigated in this study were collected along two semi-arid catenas with geologically different parent materials in South Africa's Kruger National Park (KNP; see Figure 1). The first catena (G) was located 7 km south of Skukuza in the Stevenson-Hamilton supersite, representing the southern granites of KNP, while the second catena (B) was located near Lower Sabie at the Nhlowa supersite, representing KNP's southern basalts. Both sites experience a similar mean annual precipitation (MAP; 560 mm and 610 mm, respectively), mean annual temperature (~22°C) and fire regime (mean fire return interval of 5.80 and 4.05 years) but differ markedly in regards to geology, herbivory (average herbivore biomass of 15 kg ha^−1^ and 44 kg ha^−1^), soil properties, landscape forms and vegetation (Venter et al., 2003; Smit et al., 2013). Briefly, the granitic catena is characterized by sandy soils in the upper region, clay accumulation along the slope and clayey, duplex soils with sporadic sodic patches on the footslope (Levick et al., 2010). Due to these clay dynamics, a seep zone usually establishes upslope of the clay-accumulation zones during the rainy season (November–March), so that water derived from upslope leachate exits the soil and can pond at the surface. Consequently, the bushveld vegetation also varies along the catena: The crest is dominated by broad-leaved woody species like *Combretum apiculatum*, the mid slope is characterized by *Terminalia sericea* and increasing grass cover and at the footslope, the vegetation is shaped by fine-leaved trees (mainly *Acacia* sp) and a palatable grass layer. The basaltic catena, on the other hand, is characterized by red olivine-poor, nutrient-rich soils of higher clay content and lower water conductivity, thus leading to a spatially less well-differentiated catena type. On these soils, the vegetation consists of fine-leaved tree savanna (mainly *Acacia nigrescens, Sclerocarya birrea*) with a lush grass cover (e.g., *Thermeda triandra, Panicum coloratum*).

We expected the effects of the described clay dynamics along the catena and, linked to that, spatial differentiation of the hydrological regime, to be most strongly reflected by the microbial community structure at the end of the wet season. Consequently, soil samples were taken at the end of the wet season in March 2014 using a manual soil auger (10 cm diameter) at different locations along the two catenas (Figure 1) at 5 cm depths and as four replicates each (~3 cm apart; denoted a/b/c/d). According to their topographic positions, sampling locations along the granitic catena were denoted as crest (GI), above seep zone (GII), active seep zone (GIII) and riparian zone (GIV), while the basaltic samples were denoted as crest (BI), mid slope (BII) and riparian zone (BIII).

One replicate of each sample was stored at 4°C for chemical analyses and soil respiration measurements while the other three replicates were treated immediately with RNAlater (Qiagen, Germany) to prevent loss or fragmentation of nucleic acids and were then subsequently stored at 4°C for a few days. This resulted in 21 samples for molecular biological analysis, which were stored at −80°C after centrifugation (10.000 *g*, 15 min) and removal of the supernatant, i.e., most of the applied RNAlater solution.

### Geochemical analyses

Soil extracts were generated by shaking ~2 g soil in 5 ml dH_2_O for 2 h, followed by measurement of pH and photometric determination of ammonium, nitrite and nitrate concentrations according to DEV (1975) and Grasshoff et al. (1983). Total carbon (C) and nitrogen (N) concentrations of the soil samples were measured as described previously by Klaus et al. (2013) and Schrumpf and Kaiser (2015). About 30 g of soil were freeze dried and sieved manually (2 mm). After a second sieving (0.2 mm), particle size analysis was performed by laser measurement (analysette 22 COMPACT, Fritsch, Germany).

### Microbial respiration measurements

Basal respiration (BAS), microbial biomass (C_mic_), and specific respiration (*q*O_2_) were measured by substrate-induced respiration (SIR), i.e., the respiratory response of microorganisms to glucose addition (Anderson and Domsch, 1978). Before measurement, roots were removed and soil samples were sieved (2 mm). Measurements were done using an automated O_2_ microcompensation system (Scheu, 1992). BAS of microorganisms reflected their averaged oxygen consumption rate without the addition of glucose within 10–20 h after attachment of the samples to the analysis system. Subsequently, 4 mg glucose g^−1^ soil dry weight was added as aqueous solution to the soil samples. The mean of the three lowest hourly measurements within the first 10 h was taken as the maximum initial respiratory response (MIRR). C_mic_ (μg C g^−1^) was calculated as 38 × MIRR (μl O_2_ g^−1^ soil dry weight h^−1^) according to Beck et al. (1997). Microbial specific respiration qO_2_ (μl O_2_μ g^−1^ C_mic_ h^−1^) was calculated as BAS/C_mic_ and eflects carbon use efficiency.

### DNA extraction, amplification, and clone library construction

DNA was extracted from ~0.6 g of each soil sample according to Lueders et al. (2004) dissolved in 100 μl TE buffer (pH 7.4) and stored at −80°C. Clone libraries were constructed from selected samples for *nxrB* (GI, BI), bacterial *amoA* and archaeal *amoA* (both GI, GIV, BII) genes. These genes were amplified from DNA extracts of the corresponding triplicate samples using HotStarTaq Master Mix (Qiagen, Germany). For the amplification of archaeal and bacterial *amoA* genes, primer pairs Arch-AmoAF/Arch-AmoAR (Francis et al., 2005) and AmoA-1F/AmoA-2R (Rotthauwe et al., 1997) were used according to protocols from the respective publications. *nxrB* genes affiliated with the genus *Nitrospira* were amplified using primers nxrB19f/nxrB1237r (Pester et al., 2014) and the following cycling conditions: 15 min at 95°C, 35 cycles of 94 (45 sec), 59 (45 sec), and 72°C (90 sec) and 10 min at 72°C. All these PCRs were performed in a total volume of 15 μl containing 7.5 μl of 2x HotStarTaq Master Mix (Qiagen, Germany), 1 μM of each primer, 0.67 μg/μl of BSA and 1–10 ng of DNA. Refer to supplemental material for details on respective cycling conditions and all used primer sequences (Supplementary Table 1). Denaturing Gradient Gel Electrophoresis (DGGE) was performed with archaeal *amoA* amplicons to perform fingerprinting of archaeal ammonia-oxidizing communities before cloning (see Supplementary Methods).

PCR products were checked by agarose gel electrophoresis and purified using the NucleoSpin Gel & PCR Clean-Up Kit (Macherey-Nagel, Germany) according to the manufacturer's instructions. Clone libraries were constructed from purified *nxrB* and bacterial and archaeal *amoA* amplicons using pGEM-T Easy vector and *Escherichia coli* JM109 chemically competent cells (Promega). For this purpose, PCR products were generated in triplicate per DNA extract and all PCR products from PCR replicates and replicate soil samples per site were pooled. Plasmid DNA was purified using GeneJET Plasmid Miniprep Kit (Thermo Fisher Scientific, USA) and inserts were commercially sequenced (Macrogen, Korea/The Netherlands).

### Illumina MiSeq amplicon sequencing

Illumina MiSeq amplicon sequencing targeting bacterial and archaeal 16S rRNA genes was performed on all samples using the primer combinations Bakt_805R/Bakt_341F (Herlemann et al., 2011) and Parch349F/Arch915r (Stahl and Amann, 1991; Øvreås et al., 1997), respectively. Illumina MiSeq amplicon sequencing was carried out by LGC Genomics GmbH (Berlin, Germany). The PCRs included about 5 ng of DNA extract, 15 pmol of each forward primer 341F 5′**-**NNN NNNNNNNTCCTACGGGNGGCWGCAG and reverse primer 785R 5′-NNN NNNNNNNTGACTACHVGGGTATCTAAKCC in 20 uL volume of 1 × MyTaq buffer containing 1.5 units MyTaq DNA polymerase (Bioline) and 2 μl of BioStabII PCR Enhancer (Sigma). For each sample, the forward and reverse primers had the same 10-nt barcode sequence. PCRs were carried out for 30 cycles using the following parameters: 2 min 96°C predenaturation; 96°C for 15 s, 50°C for 30 s, 70°C for 90 s. DNA concentration of amplicons of interest was determined by gelelectrophosesis. About 20 ng amplicon DNA of each sample were pooled for up to 48 samples carrying different barcodes. If needed PCRs showing low yields were further amplified for 5 cycles. The amplicon pools were purified with one volume AMPure XP beads (Agencourt) to remove primer dimer and other small mispriming products, followed by an additional purification on MinElute columns (Qiagen). About 100 ng of each purified ampliconpool DNA was used to construct Illumina libraries using the Ovation Rapid DR Multiplex System 1–96 (NuGEN). Illumina libraries were pooled and size selected by preparative gelelectrophoresis. Sequencing was done on an Illumina MiSeq using V3 chemistry (Illumina).

### Sequence analysis

Sequences obtained from *nxrB* and *amoA* clone libraries were transformed to amino acid sequences and analyzed using manually generated reference protein alignments in ARB (Ludwig et al., 2004). Nucleic acid-level alignments were created according to the deduced amino acid alignments, followed by the generation of nucleic acid level distance matrices in ARB and clustering of sequences to Operational Taxonomic Units (OTUs) using the average neighbor algorithm implemented in Mothur (Schloss et al., 2009). The following distance cutoffs were used: 0.15 for archaeal *amoA* genes (Pester et al., 2012), 0.20 for bacterial *amoA* genes (Purkhold et al., 2000) and 0.05 for *nxrB* genes (Pester et al., 2014). Closest relatives were determined based on a BLAST (http://blast.ncbi.nlm.nih.gov/Blast.cgi) search of sequences representative of each OTU. Phylogenetic trees including sequences representative of each OTU were constructed (neighbor-joining; 1000 bootstrap replicates) in ARB based on the respective protein reference alignments.

Illumina MiSeq amplicon sequencing was performed for bacterial and archaeal 16S rRNA genes obtained from all soil samples. The software Mothur was used to analyze all obtained reads (Schloss et al., 2009). Bacterial and archaeal 16S rRNA gene reads were analyzed according to MiSeq SOP (http://www.mothur.org/wiki/MiSeq_SOP; Kozich et al., 2013). Briefly, the paired-end reads were assembled and contigs of incorrect lengths (>554 bp and >440 bp, respectively), containing ambiguous bases and/or over 8 detected polymers were removed. The resulting bacterial and archaeal 16S rRNA gene sequences were aligned to the SILVA reference database (v119; Quast et al., 2013). Potential chimeric sequences were detected and removed using the uchime algorithm implemented in Mothur. The remaining sequences were taxonomically classified and clustered into operational taxonomic units (OTUs) using a 0.03 distance cutoff. By using OTU group representative sequences, the archaeal taxonomy affiliations were then manually checked and re-assigned when needed based on additional reference sequences from uncultured or newly described archaeal taxa.

Several authors, e.g., Schloss et al. (2011), have suggested to normalize the number of sequence reads per sample for diversity analyses to allow a better comparability of different data sets. Moreover, we removed singleton OTUs which may originate from sequencing errors and thus introduce artificial diversity. In brief, we normalized sequence reads of all samples to the same number of sequence reads using the sub.sample command in Mothur. In order not to lose too much sequence information across samples, a few samples with too low read numbers were excluded from further analysis (GII_c, GIIIc, GIV_b/c, BI_a, BIII_c for bacteria; GII_b, GIV_b, BI_b/c, BIII_c for archaea, see Supplementary Table 2). Subsequently, we removed singleton OTUs using the remove.rare command in Mothur. The results of sequence analysis and alpha diversity before subsampling and singleton removal are presented in Supplementary Table 4. The taxonomic classification of bacterial and archaeal 16S rRNA gene sequence reads before normalization and removal of singleton OTUs is shown in Supplementary Figures 7 and 8, respectively, also including replicate samples which were excluded during the subsampling procedure.

All analyzed clone sequences were submitted to the GenBank database under the accession numbers KX160221–KX160439. Bacterial and archaeal 16S rRNA amplicon Illumina sequencing raw data were deposited in the European Nucleotide Archive database under the study accession number PRJEB13568.

### Quantitative PCR

To determine the abundances of bacterial and archaeal 16S rRNA genes and *amoA*, quantitative PCR was performed on a Mx3000P cycler (Agilent Technologies, USA) using Maxima SYBR Green qPCR Mastermix (Thermo Fisher Scientific Inc., USA). 16S rRNA genes were amplified using primer pairs Bac8Fmod/Bac338Rabc (Daims et al., 1999; Loy et al., 2002) and Arch806F/Arch958R (DeLong, 1992; Takai and Horikoshi, 2000), according to cycling conditions and standards described previously by Herrmann et al. (2012). Bacterial and archaeal *amoA* genes were amplified with primer pairs AmoA-1F/AmoA-2R (Rotthauwe et al., 1997) and Arch-AmoAF/Arch-AmoAR (Francis et al., 2005) under cycling conditions as in Herrmann et al. (2008).

Standard curves were generated using serial dilutions of representative plasmid mixtures with environmental sequences of the respective genes, chosen from respective clone libraries obtained in this study in the case of functional genes or obtained as described in Herrmann et al. (2012) in the case of 16S rRNA genes. All qPCR runs showed *R*^2^ ≥ 0.99 and efficiencies in the range of 80–98%. Potential PCR inhibition was tested for by 10 times dilution series of representative samples, which yielded similar qPCR efficiencies as obtained for the standard dilution series. In cases where the gene abundances per reaction were below the quantification limit (i.e., 50 copies/reaction), a maximum estimate of gene abundances per g soil was calculated based on this quantification limit. Fractions of microorganisms harboring *amoA* genes within the total microbial population were estimated from respective qPCR results by using the following correction factors to account for the possibility of multiple gene copies per genome: 4.02 for the bacterial 16S rRNA gene and 1.63 for the archaeal 16S rRNA gene (https://rrndb.umms.med.umich.edu/; Stoddard et al., 2015) and 1.0 for the archaeal *amoA* gene (Leininger et al., 2006).

### Statistical analysis

Correlations between gene abundances and chemical soil properties were analyzed using Spearman rank correlation coefficient in PAST (V3.07; Hammer et al., 2001) and results were viewed as significant if a two-tailed level of significance of at least 0.05 was reached. Bacterial community structure as determined by Illumina sequencing (i.e., 16S rRNA reads per OTU and sampling location) as well as distribution of AOA-*amoA* sequence types based on DGGE band patterns was also combined with chemical soil properties and subjected to canonical correspondence analysis in PAST. For this purpose, read numbers obtained per OTU from replicate samples were averaged and average values of less than one read were set to 1. For CCA based on DGGE band patterns, results of presence/absence matrices of triplicate samples per site were combined by setting the presence to “1” if a band was present in at least one of the three replicates. Differences in gene abundances between sites were tested using Mann-Whitney-U-test in SPSS 21.

## Results

### Bacterial soil communities in oligotrophic, geochemically heterogeneous savanna soils

The granitic savanna soils were dominated by sand-sized particles, were moderately acidic (pH_G_ = 5.5 ± 0.58) and showed mostly low nutrient contents (means: *N* = 0.05%; *C* = 0.45%; 13.87 ± 9.63 μg g^−1^ ${\text{NO}}_{3}^{-}$; 4.27 ± 5.34 μg g^−1^ ${\text{NH}}_{4}^{+}$). The sampling location GII, i.e., above the active seep zone at the time of sampling, exhibited a particularly distinct geochemistry with the most acidic soils (pH_GII_ = 4.7), along with the highest concentrations of ammonium (i.e., 12.04 μg g^−1^) observed along the granitic catena and the lowest clay, nitrate, organic C and N contents across samples from both catenas (Table 1). Both the fine particle fraction and general nutrient content increased slightly along the granitic catena, while the basaltic catena showed less spatial differentiation of soil properties along the slope. Basaltic soil samples contained higher silt and clay fractions, were only slightly acidic (pH_B_ = 6.2 ± 0.05) and exhibited on average five times higher soil N, C and nitrate contents than the granitic soils with the highest ammonium concentration again being detected at the mid-slope position, i.e., in this case 33.23 μg g^−1^ at BII. Nitrite concentrations were generally low at both catenas (range: 0–5.35 μg g^−1^, data not shown).

**Table 1:** **Geochemical soil properties and microbial respiration measurements**.

**Sampling sites**		**Soil properties**					**Microbial respiration**		
**location**	**sample code**	**pH**	**${\text{NO}}_{3}^{-}$**	**${\text{NH}}_{4}^{+}$**	**C**	**N**	**BAS**	**C_mic_**	**qO_2_**
**GRANITIC CATENA (G)**									
crest	GI	5.4	15.5	3.54	0.36	0.05	1.59	95.85	0.0166
above seep zone	GII	4.7	0.0	12.04	0.32	0.04	1.37	68.82	0.0199
active seep zone	GIII	6.1	17.9	0.83	0.50	0.06	1.38	79.57	0.0173
riparian	GIV	5.8	22.0	0.69	0.63	0.06	1.52	122.28	0.0125
**BASALTIC CATENA (B)**									
crest	BI	6.2	7.1	9.97	1.80	0.14	1.57	186.52	0.0084
mid slope	BII	6.2	121.9	33.23	3.01	0.27	1.71	398.66	0.0043
riparian	BIII	6.3	17.6	18.51	2.78	0.21	1.61	207.16	0.0078

*Soil pH, nitrate and ammonium concentrations [μg g^−1^], total C and N content [%], basal respiration rate [BAS; μl O_2_g^−1^soil dw h^−1^], microbial biomass [C_mic_; μg C_mic_ g^−1^ soil dw] and microbial specific respiration [qO2; μl O_2_μg^−1^C_mic_ h^−1^] are shown per soil sample. Soil samples' catena of origin, sampling location and resulting sample codes are also denoted. G, granitic catena; B, basaltic catena*.

**Figure 2:**
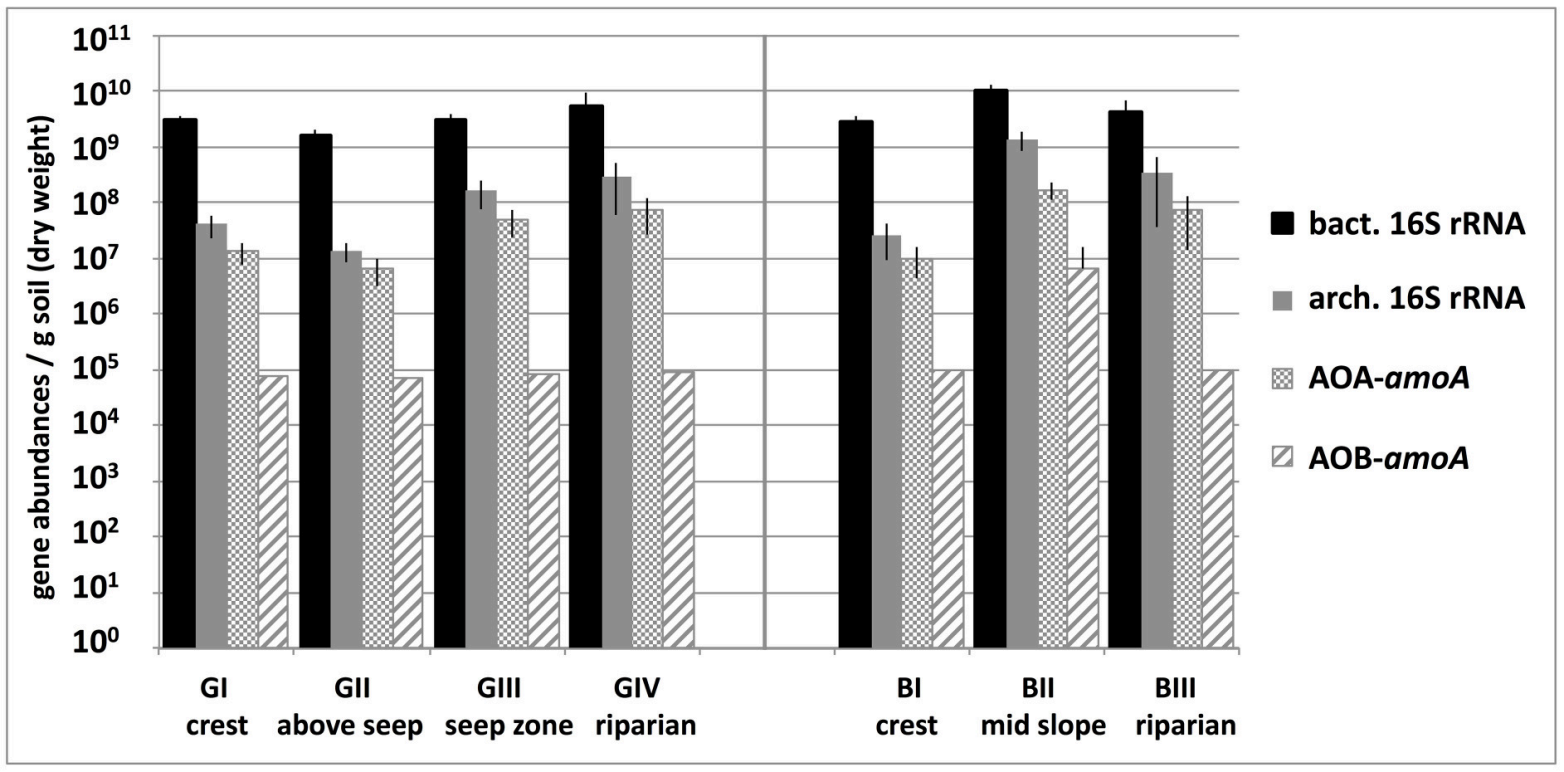
**Abundances of bacterial and archaeal 16S rRNA genes and archaeal and bacterial *amoA* genes (AOA-*amoA*, AOB-*amoA*) per gram soil in savanna soil samples taken from 5 cm depth at different locations along the granitic (GI-GIV) and the basaltic (BI-BIII) catena**. Bars represent mean abundances of replicate samples, each measured in triplicate qPCR reactions, and respective standard deviations. Abundances of bacterial *amoA* genes were below the quantification limit (50 genes per qPCR reaction) in all samples except BII and maximum estimates based on this limit are shown.

Bacterial 16S rRNA genes showed abundances around 4 × 10^9^ copies g^−1^ soil, which increased slightly along the granitic catena, and were highest in basaltic soils and significantly lower at GII compared to all other locations (Figure 2). Basal respiration rates were similar in all tested samples (mean BAS = 1.54 μl ± 0.12 O_2_g^−1^soil dw h^−1^), while microbial biomass (C_mic_) varied between the two catenas and between sampling sites along each catena, reaching a maximum of 398.66 μg C_mic_ g^−1^soil dw at the basaltic mid-slope location (BII) and a minimum of 68.82 μg C_mic_ g^−1^ soil dw above the granitic seep zone (GII). Carbon use efficiency (qO_2_) was also higher in the basaltic soils (mean qO_2B_ = 0.0068 μl O_2*m*_ g^−1^ C_mic_ h^−1^) than in the granitic soils and especially low at GII with qO_2GII_ = 0.0199 μl O_2_μg^−1^ C_mic_ h^−1^ (Table 1).

**Figure 3:**
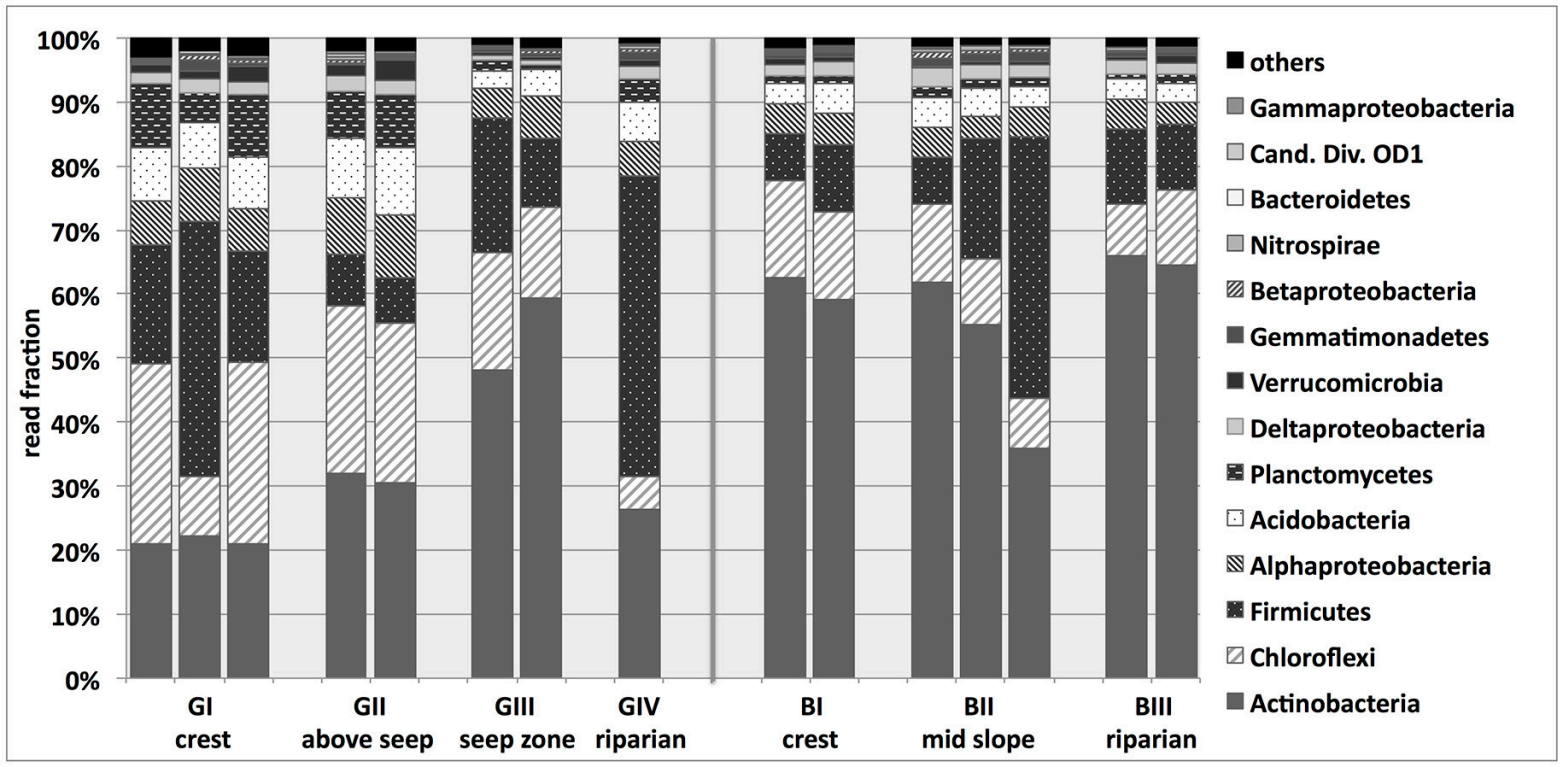
**Phlyum-level taxonomic classification of sequences obtained from bacterial 16S rRNA gene-targeted Illumina sequencing of savanna soil samples taken from 5 cm depth at different locations along the granitic (GI-GIV) and the basaltic (BI-BIII) catena**. Cand. Div., Candidate Division. For some samples, replicates had to be excluded from further analysis of the normalized data set due to low read numbers. Results based on the non-normalized sequence data, including more soil replicates, are presented in Supplementary Figure 7.

Illumina sequencing targeting bacterial 16S rRNA genes resulted in 634 to 979 observed bacterial OTUs per sample determined at 97% sequence identity (Supplementary Table 2) and revealed that the investigated bacterial soil communities were dominated by *Actinobacteria* (21–66% of sequence reads per sample), *Chloroflexi* (5–28%) and *Firmicutes* (7–47%; Figure 3). *Planctomycetes, Acidobacteria, Alpha*- and *Deltaproteobacteria* showed lower read frequencies >1%. The granitic upslope soils (GI, GII), appeared to harbor the highest phylum-level diversity, with e.g., *Planctomycetes* and *Acidobacteria* accounting for the highest read fraction across the whole transect at that location. The relative fraction of sequence reads affiliated with *Actinobacteria* increased along the granitic catena, reaching levels of around 50% at site GIII. *Chloroflexi* -associated reads, on the other hand, decreased in relative abundance along the granitic catena, while no clear trend could be observed for Firmicutes. The bacterial community composition at sites BI-III was very similar to the granitic downslope sites with even higher fractions of sequence reads affiliated with *Actinobacteria* (36–66% of sequence reads) and lower fractions of *Chloroflexi* (8–15%) (Figure 3).

For both the granitic and the basaltic soils, most detected *Actinobacteria* belonged to the classes *Actinobacteria* (41%; predominantly Frankiales) or *Thermoleophilia* (41%). Most of the *Firmicutes*-affiliated reads belonged to *Bacilli* (91%) and *Clostridia* (6%) and most of the *Chloroflexi*-affiliated reads were assigned to *Ktedonobacteria* (39%), group TK10 (20%) and JG37-AG-4 (12%). The majority of *Acidobacteria* reads belonged to the subgroups 1, 3, 4, and 6 or remained unclassified. On the whole, the classes *Actinobacteria, Thermoleophilia* and *Bacilli* made up the majority of the bacterial community at most of the sampling sites, with *Ktedonobacteria* being additionally abundant and almost exclusively detected at GI and GII (Supplementary Figure 1).

**Figure 4:**
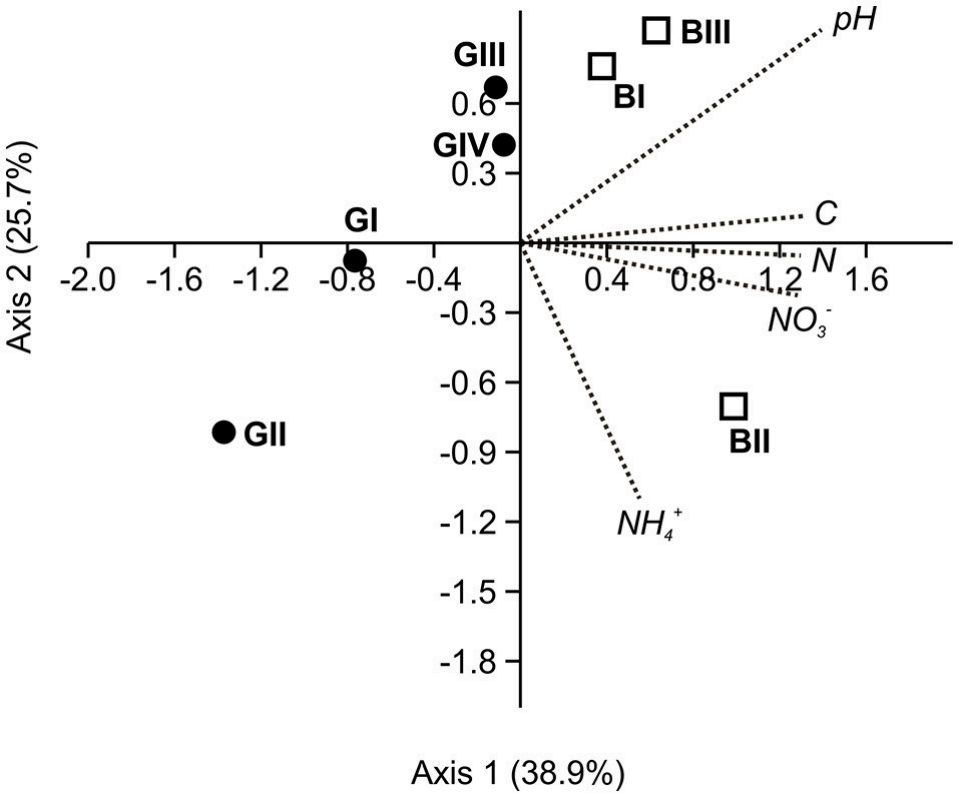
**Biplot from canonical correspondence analysis (CCA) of five measured environmental variables (ammonium concentration, nitrate concentration, total C, total N, soil pH) and the bacterial 16S rRNA gene-based community structure in soil samples from two catenas, detected as average read numbers of OTUs determined at 97% sequence identity on nucleic acid-level per sampling location**. For this purpose, samples were normalized to the same number of total reads prior to analysis. Samples from the granitic and the basaltic catena are shown as filled circles (GI-GIV) and open squares (BI-BIII), respectively.

According to results from correlation analysis using Spearman rank correlation coefficient, soil pH and soil C and N content and nitrate concentration were significantly correlated with C_mic_, while the latter three parameters also showed significant correlations with BAS (Table 2). Additionally, a canonical correspondence analysis performed with five environmental variables (ammonium and nitrate concentrations, total C and N content, soil pH) and the bacterial 16S rRNA-derived bacterial community structure based on OTUs determined at 97% sequence identity (read frequencies of OTUs, averaged from triplicate soil samples per sampling location) revealed a clear separation of soil samples obtained from the granitic and basaltic catena. This separation mostly occurred along axis 1, showing a higher heterogeneity in the granitic samples than the basaltic samples, which clustered closer together (Figure 4). Axis 1 accounted for 38.9% of the observed variability in the bacterial community structure and was most strongly correlated with soil pH (score = 0.92) but also correlated with total C and N content and nitrate (scores = 0.88; 0.87; 0.86, respectively). Additionally, separation of the granitic above seep location (GII) from all other sampling points along both axes was observed. Axis 2 explained 25.7% of the observed variation and was negatively correlated with ammonium content (score = −0.73) but showed only weak correlations with the other tested environmental variables (scores < 0.60).

**Table 2:** **Correlations (Spearman's rank correlation coefficients) between abundances of archaeal *amoA* (AOA-*amoA*), archaeal and bacterial 16S rRNA genes, soil chemical parameters (soil pH, ammonium and nitrate concentrations, total C and N content) and respiration-based parameters (BAS, basal respiration; C_mic_, microbial biomass) along the granitic and basaltic catena**.

	**${\text{NO}}_{3}^{-}$**	**${\text{NH}}_{4}^{+}$**	**%C**	**%N**	**BAS**	**C_mic_**	**AOA-*amoA***	**bact. 16S rRNA**	**arch. 16S rRNA**
pH	0.93^**^	0.18	0.93^**^	0.93^**^	0.71	0.86^*^	0.71	0.50	0.71
${\text{NO}}_{3}^{-}$		0.21	1^***^	1^***^	0.79^*^	0.96^***^	0.79^*^	0.68	0.79^*^
${\text{NH}}_{4}^{+}$			0.21	0.21	0.43	0.29	0.14	0.07	0.14
%C				1^***^	0.79^*^	0.96^***^	0.79^*^	0.68	0.79^*^
%N					0.79^*^	0.96^***^	0.79^*^	0.68	0.79^*^
BAS						0.89^*^	0.71	0.71	0.71
C_mic_							0.75	0.71	0.75
AOA-*amoA*								0.93^**^	1^***^
bact. 16S rRNA									0.93^**^

Two-tailed levels of significance are indicated as follows:

* *0.05*,

** *0.01*,

*** *0.001*.

### Composition of archaeal communities in savanna soils

**Figure 5:**
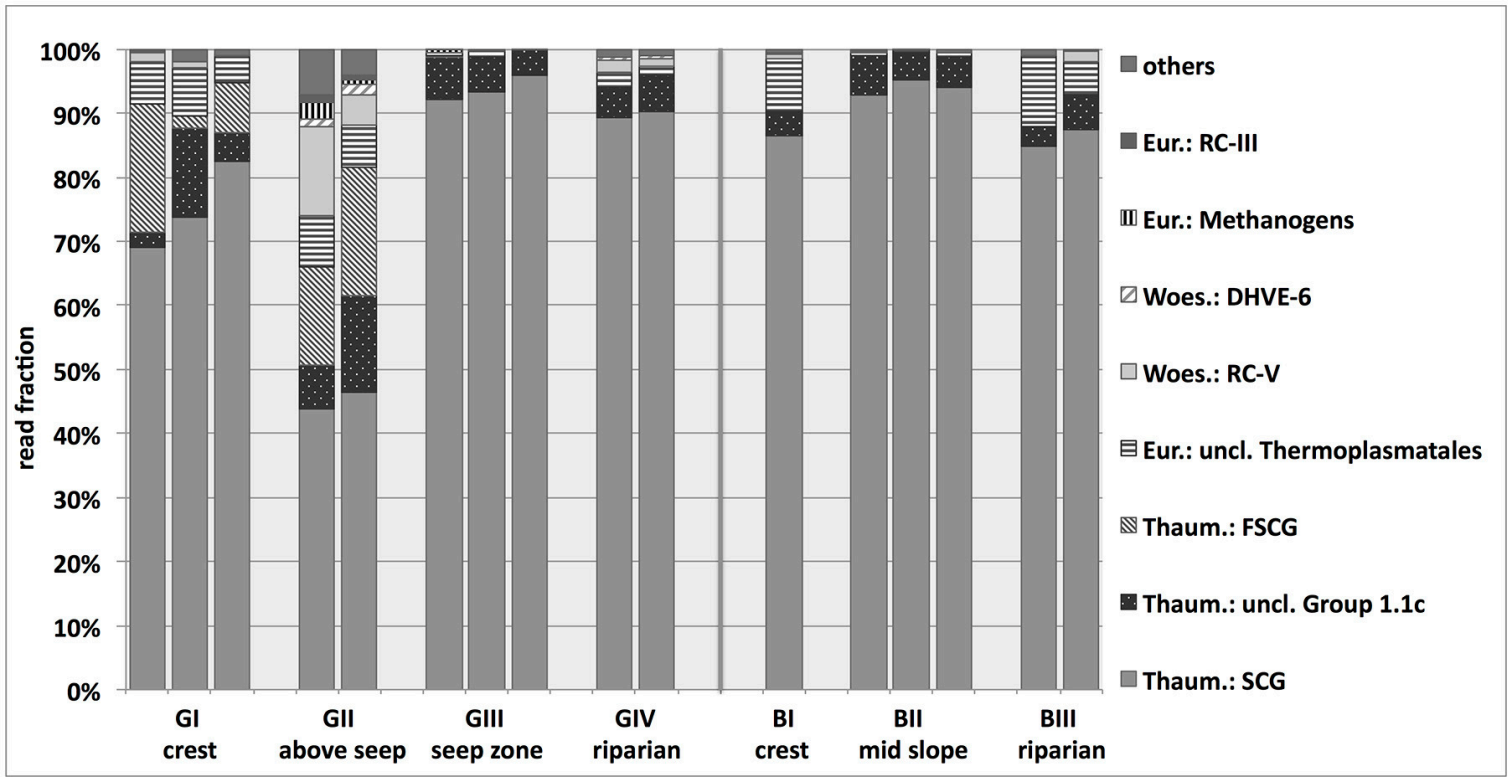
**Classification of sequences obtained from archaeal 16S rRNA gene-targeted Illumina sequencing of savanna soil samples taken from 5 cm depth at different locations along the granitic (GI-GIV) and the basaltic (BI-BIII) catena**. Eur, Euryarchaeota; RC, Rice Cluster; Woes, Woesearchaeota; DHVE, Deep Sea Hydrothermal Vent Euryarchaeota Group; Thaum, Thaumarchaeota; uncl, unclassified; (F)SCG, (Forest) Soil Creanarchaeota Group. For some samples, replicates had to be excluded from further analysis of the normalized data set due to low read numbers. Results based on the non-normalized sequence data, including more soil replicates, are presented in Supplementary Figure 8.

Archaeal 16S rRNA gene abundances varied around 3 × 10^8^ copies g^−1^ soil and suggested that *archaea* accounted for 7% or 14% of the total microbial community on average at the granitic and basaltic catena, respectively (range across all sites: 2–24%; see Figure 2). The relative fraction of *archaea* in the total microbial communities increased in the soils of the downslope locations at both catenas. Illumina MiSeq amplicon sequencing resulted in 12 to 51 observed archaeal OTUs and revealed that almost all obtained archaeal 16S rRNA reads belonged to *Euryarchaeota* or *Thaumarchaeota*, although a few taxa from *Woesearchaetoa* were also detected (Figure 5). The most abundant and ubiquitous archaeal group was the thaumarchaeal Soil Crenarchaeotic Group (SCG), which made up 43–96% of all reads at the different sampling locations. A BLAST search conducted with representative sequences of the 20 most abundant OTUs falling into this SCG classification revealed high levels of sequence identity (96% on average) with cultured representatives of the ammonia-oxidizing genus *Nitrososphaera*. The only other group found across all samples was the thaumarchaeal Group 1.1c, while the thaumarchaeal Forest Soil Crenarchaeotic Group (FSCG), the woesearchaeal Rice Cluster V Group (RC-V) and euryarchaeal unclassified *Thermoplasmatales* also accounted for a high fraction of sequence reads in some samples (20, 14, and 11%, respectively). Notably, FSCG appeared most dominant in the granitic upslope soils, especially above the granitic catena's seep zone (GII). Reads belonging to RC-V were found in several samples and were also particularly abundant at site GII. Other groups with read frequencies higher than 1% in some samples were assigned to the Deep sea Hydrothermal Vent Euryarchaeota Group 6 (DHVE-6), the Rice Cluster III Group (RC-III) and different euryarchaeal methanogenic groups. The latter three of these groups were almost exclusively found above the seep zone (GII), where e.g., the methanogenic *archaea* together reached a maximum abundance of 2.7%. In summary, a clear variation of archaeal community structure was observed along the granitic catena, with more diverse archaeal communities occurring in the upslope soils above the seep zone (GI, GII). In contrast, the archaeal communities at the granitic and basaltic downslope positions (GIII, GIV, BII) were largely dominated by *Thaumarchaeota* affiliated with AOA. Additionally, GII was the only site where considerable abundances of methanogenic *archaea* were detected and the site with highest abundances of RC-V, DHVE-6, RC-III and methanogen affiliated 16S rRNA reads.

### Microbial communities potentially involved in nitrification in savanna soils

Archaeal *amoA* gene abundances varied between 6.5 × 10^6^ and 1.7 × 10^8^ genes g^−1^ soil and outnumbered bacterial *amoA* genes by at least two orders of magnitude at almost all sampling locations. Furthermore, qPCR-based gene abundances suggested that AOA made up 1.4–6.9% of the total microbial community and 21–77% of the archaeal community. Abundances of bacterial *amoA* genes, on the other hand, were below the method's quantification limit (ca. 7 × 10^4^ copies g^−1^ soil) in all samples except BII where 6 × 10^6^ bacterial *amoA* genes g^−1^ soil were detected. Both archaeal 16S rRNA and *amoA* gene abundances increased along the granitic catena (Figure 2) and showed significantly higher numbers at BII compared to all the other sites, which was related to their positive significant correlation with soil nitrate concentration and soil C and N contents (see Table 2).

In order to gain further insight into the nitrifying microbial communities in savanna soils, the community composition of bacterial and archaeal ammonia oxidizers (AOB, AOA) and nitrite oxidizing bacteria (NOB) were investigated using functional gene-based approaches. Band patterns from an AOA-*amoA*-based DGGE and the reported environmental parameters were used in a CCA, which revealed patterns similar to those observed for the 16S rRNA-derived bacterial community structure, i.e., a separation of granitic and basaltic samples, high similarity among the latter sample group and a distinct character of the microbial community at GII (Supplementary Figures 2, 3). Cloning and sequence analysis of archaeal and bacterial *amoA* genes revealed a much higher diversity for AOA than AOB: Using distance cutoffs of 0.15 and 0.20 on nucleic acid level, respectively, 12 AOA-OTUs were observed, while all bacterial *amoA* sequences fell into one single OTU (Supplementary Table 3). The detected AOB-OTU was affiliated with the *Nitrosospira* lineage and grouped together with other sequences of uncultured organisms from soils and sediments (Supplementary Figure 4). All the detected AOA-OTUs were part of the *Nitrososphaera* cluster where they clustered together with environmental sequences from other soil habitats (Supplementary Figure 5). Several OTUs were observed that exclusively occurred in either the granitic crest soils (GI) or the granitic riparian zone (GIV) and one OTU was also exclusively detected in basaltic soils.

Based on the results from Illumina sequencing targeting bacterial 16S rRNA genes, only very few reads were classified as phylotypes belonging to the polyphyletic nitrite-oxidizing bacteria. The most abundant NOB group detected was *Nitrospira* sp., which accounted for 0.1% of all obtained bacterial 16S rRNA reads across sites. The two clone libraries constructed for the *Nitrospira* sp.-derived *nxrB* gene resulted in a total of 16 OTUs (distance cutoff: 0.05 on nucleic acid level), which were affiliated with *Nitrospira moscoviensis* (identities: 89–93%) and several other *Nitrospira*-related sequences (Supplementary Figure 6). Interestingly, several *nxrB*-OTUs included only sequences from either the basaltic or the granitic catena soil samples.

## Discussion

### Oligotrophic savanna soils harbor distinct bacterial communities

Our results clearly showed that the nutrient-poor conditions in the South African savanna soils resulted in a community structure which is distinctly different from those usually found in temperate grassland soils, and was dominated by Actinobacteria, Chloroflexi and Firmicutes, with an unusually high fraction of archaea.

The considerable nutrient limitations of the investigated savanna soils were clearly confirmed by our geochemical measurements, consistently falling at the low end of soil N or C content ranges reported in global grassland surveys, which found N and C contents up to 7 or 38 times higher than in KNP granitic or basaltic soils, respectively (Will et al., 2010; Catão et al., 2013; Meyer et al., 2013; Legay et al., 2014; Leff et al., 2015; Prober et al., 2015). While the very oligotrophic granitic soils also exhibited lower nitrate and ammonium concentrations than other grasslands soils, the basaltic soils' values were more similar to other habitats (e.g., temperate fields, semiarid steppes, subalpine pastures; Pereira e Silva et al., 2012; Hartmann et al., 2013; Augustine et al., 2014). The observed geochemical differences between the two investigated catenas, i.e., granitic soils being more sandy and acidic, while basaltic soil samples contained higher silt, clay and nutrient contents, generally agree with previously described effects of the different bedrock materials on KNP soil properties (Scholes, 1990; Venter et al., 2003; Coetsee et al., 2012). Similarly, the KNP's low soil nutrient contents confirm previous reports (Aranibar et al., 2003; Coetsee et al., 2012) of other African savanna soils (Bernhard-Reversat, 1996; Ndiaye et al., 2004; Cech et al., 2008; Braker et al., 2015).

Regardless of the nutrient limitations, the detected general bacterial 16S rRNA gene abundances and BAS were similar to results from other temperate soil environments (Kemnitz et al., 2007; Sims et al., 2012; Eisenhauer et al., 2013; Sheng et al., 2013). Heterotrophic microbial biomass C_mic_ was typically low, e.g., compared to Steinauer et al. (2015), and only exhibited higher values in those samples with higher nutrient availability (i.e., GIV, BI-III), which agrees with the slightly higher bacterial abundances detected in those soil samples. Here, the observed lower qO_2_ values also indicated a higher carbon use efficiency of (or less stress for) the soil heterotrophic microbial community (e.g., Araujo et al., 2012). Supported by the CCA results and significant positive correlations between soil C and N content and microbial parameters, our results clearly demonstrate the strong effect of nutrient availability on bacterial abundances and (heterotrophic) activities in the investigated nutrient poor savanna soils.

Apparently, the nutrient poor soil conditions were also a strong driver of soil microbial community structure and the dominant taxonomic groups. The most abundant bacterial phyla in soils from both catenas were *Actinobacteria, Chloroflexi* and *Firmicutes*, while *Planctomycetes, Acidobacteria* and *Alpha*- and *Deltaproteobacteria* were the most frequent phyla among those with lower relative abundances. This community structure differs markedly from the composition typically observed in other soil ecosystems: Several large surveys have repeatedly identified *Acidobacteria* and (*Alpha*-) *Proteobacteria* as the two dominant bacterial phyla in soils, often accompanied by *Actinobacteria* as another abundant group (Janssen, 2006; Lauber et al., 2009; Nacke et al., 2011; Prober et al., 2015). Each DNA extraction method may come along with a bias, and we cannot rule out that the microbial community structure observed in the savanna soils may have been influenced by the DNA extraction method chosen. However, other studies using the same nucleic acid extraction method have previously reported more commonly expected soil community structures, including high fractions of *Proteobacteria* (e.g., Dibbern et al., 2014), rendering it unlikely that the low fraction of *Beta- and Gammaproteobacteria* in the soils of the granitic and basaltic catena was mainly caused by DNA extraction bias. Potential common savanna-specific factors underlying the observed distinct community structure cannot fully be identified at this point because comparative data on other savanna soil bacterial communities is still relatively scarce and inconclusive (e.g., Araujo et al., 2012; Rampelotto et al., 2013; Huber, 2015). Notably, soils with similarly high abundances of *Firmicutes* and/or *Chloroflexi* are typically found in rather oligotrophic environments, e.g., geothermal soils (Gagliano et al., 2015), post-mining reconstructed soils (de Quadros et al., 2016), Antarctic soils (Kim et al., 2014; Ji et al., 2015) or alpine wet tundras (Costello and Schmidt, 2006), again pointing to the nutrient-poor conditions in the KNP soils as a strong driver of soil bacterial community structure.

Thus, the dominance of *Actinobacteria, Chloroflexi* and *Firmicutes* in the KNP soils might be due to these phyla being well-adapted to typical savanna nutrient and water limitations. Firstly, *Actinobacteria* can be especially resistant to heat and aridity (Araujo et al., 2012; Neilson et al., 2012) and *Chloroflexi* have often been found in high abundances in extreme environments characterized by water or nutrient stress (Freeman et al., 2009; Weber and King, 2010; Neilson et al., 2012). Likewise, the predominant *Firmicutes* taxa *Bacilli* and *Clostridia* contain well-described groups with spore forming abilities and heat tolerance (de Vos et al., 2009), which is likely an advantageous strategy in the KNP savannas' marked wet/dry seasonality and also with regard to the regular wildfires occurring in savannas, as recently also discussed by Chávez-Romero et al. (2016). The fact that *Actinobacteria* appeared to increase in unison with soil nutrient contents, such as N and C content across both catenas, while relative abundances of *Chloroflexi* decreased, additionally points to nutrient availability driving the bacterial community structure and agrees well with the copiotrophic hypothesis (Fierer et al., 2007), which predicts that fast-growing soil bacteria (copiotrophs) prefer nutrient rich environments, while slower growing oligotrophs would thrive in oligotrophic soils. Many actinobacterial groups show a copiotrophic lifestyle (Leff et al., 2015; Chávez-Romero et al., 2016), and are known as heterotrophic decomposers of soil organic matter (Janssen, 2006; Ventura et al., 2007; Větrovský and Baldrian, 2015) or N_2_ fixing bacteria (e.g., orders *Frankiales* and *Pseudonocardiales*), which would be expected in the rhizosphere of *Acacia* sp. found in the catena's downslope areas but not below *Combretum* sp. and *Terminalia* sp. at the upper granitic catena (Palo et al., 1993). Similarly, several studies have suggested an oligotrophic lifestyle for *Chloroflexi* (Ding et al., 2013; Wang et al., 2016). Finally, the copiotrophic hypothesis might also explain the observed low relative abundances of *Proteobacteria*. Especially the classes of *Beta*- and *Gammaproteobacteria* have been shown to profit from increasing nutrient or, more specifically, carbon availability and are thus generally classified as copiotrophic bacterial taxa (Fierer et al., 2007; Ding et al., 2013; Leff et al., 2015; Wang et al., 2016). Consequently, the nutrient-poor conditions in the savanna soils of Kruger National Park may have restricted the presence of copiotrophic *Betaproteobacteria*, and their low relative abundances could be interpreted as a result of being outcompeted by oligotrophs under stress conditions of low resource concentrations, as suggested by Fierer et al. (2007).

Since many of the dominant bacterial subphyla observed in the KNP soils currently lack detailed descriptions, their adaptations to the described soil conditions might follow similar strategies but remain rather speculative at this point. For example, the detected acidobacterial subdivisions 1, 3, 4, and 6 are relatively common in soils, partially oligotrophic and differentially influenced by soil pH (Lauber et al., 2009; Huber, 2015), but their ecophysiology is still unclear.

### *Archaea* dominate the ammonia-oxidizing communities in oligotrophic savanna soils

High relative archaeal abundances of ≥ 10% as observed at sites GIII, GIV, and BII, BIII in this study are typically only reported from other nutrient limited environments (e.g., acidic forest soils, high-elevation meadows, semi-arid shrublands; Kemnitz et al., 2007; Bates et al., 2011). Such oligotrophic soils might favor *archaea* since they are thought to be evolutionarily well-adapted to nutrient-poor environments and to the resulting energy stress (Valentine, 2007). Nonetheless, archaeal 16S rRNA gene abundances were positively correlated with nutrient contents, showing that also the archaeal community was positively affected by higher nutrient availability in the KNP savanna soils.

The majority of archaeal 16S rRNA Illumina sequence reads belonged to *Thaumarchaeota*, more specifically the ammonia-oxidizing genus Cand. *Nitrososphaera* or other unclassified members of the SCG, which clearly dominated the archaeal communities across all samples. Our results agree relatively well with the information available on archaeal communities in other soils, i.e., a general dominance of mesophilic *Crenarchaeota*, more recently classified as *Thaumarchaeota* (Pester et al., 2011), along with minor fractions of *Euryarchaeota* (Kemnitz et al., 2007; Timonen and Bomberg, 2009; Bates et al., 2011). Similar results were also obtained from Brazilian Cerrado savanna soils (Catão et al., 2013; de Castro et al., 2016). The second most abundant archaeal group FSCG was mainly found at the slightly more acidic sites (GI, GII), which is in accordance with previous findings that their abundance was regulated (i.e., decreased) by increasing pH in forest soils (Lehtovirta et al., 2009).

The strong dominance of ammonia-oxidizing thaumarchaeota within the archaeal communities was further supported by the numerical predominance of archaeal *amoA* gene abundances (~5 × 10^7^ copies g^−1^ soil) relative to bacterial *amoA* gene abundances (mostly ≤ 7 × 10^4^ copies g^−1^ soil). To our knowledge, a predominance of *archaea* within the ammonia-oxidizing communities has not yet been described for savanna soils but is well known for various other soil environments (e.g., Erguder et al., 2009; Herrmann et al., 2012; Sims et al., 2012; Ding et al., 2015; Ning et al., 2015). AOA tend to dominate over AOB in acidic soils and can also thrive at much lower substrate concentrations than AOB due to a higher substrate affinity (Leininger et al., 2006; Martens-Habbena et al., 2009; Zhang et al., 2012; Yao et al., 2013). Consequently, the dominance of AOA observed in the KNP soils might be related to the moderately acidic soil conditions and relatively low ammonium concentrations. However, similar to the total archaeal population, the abundances of archaeal *amoA* genes correlated with soil nitrate, C and N contents, suggesting that the increased nutrient availability at some sampling sites supported higher abundances of ammonia oxidizers without shifting the competitive advantage toward AOB. Even though all detected AOA-OTUs were affiliated with the *Nitrososphaera* cluster, 9 out of the 12 OTUs were only found in either granitic or basaltic soils, suggesting some niche differentiation of AOA across the two geologically contrasting catenas, which is in line with the described highly diverse ecophysiology of *Thaumarchaeota* (reviewed in Hatzenpichler, 2012; Stahl and de la Torre, 2012).

Only very low fractions of the bacterial soil community in the investigated samples were found to belong to known NOB phylotypes but still exhibited a considerable diversity. For the most abundant group of the *Nitrospira*-related NOB, 17 OTUs were detected based on *nxrB* gene sequencing, 95% sequence identity cut-off for OTU assignment, which were phylogenetically affiliated with *Nitrospira moscoviensis* and several other uncultured *Nitrospira* sp. Similar to AOA, these OTUs were specific to either the basaltic or the granitic catena, indicating that diversity within the group might be based on ecophysiological traits supporting niche differentiation, which agrees with the higher ubiquity and phylogenetic diversity of *Nitrospira*-type NOB compared to *Nitrobacter*-type NOB reported by other authors (Freitag et al., 2005; Xia et al., 2011; Pester et al., 2014). The observed predominance of *Nitrospira*- over *Nitrobacter*-type NOB as inferred from their representations among 16S rRNA gene Illumina reads might be related to the low soil contents of inorganic nitrogen, since *Nitrobacter*-related NOB might show higher growth rates and lower substrate affinities than *Nitrospira*-related NOB (Attard et al., 2010; Xia et al., 2011; Wertz et al., 2012).

### Potential effects of catena dynamics on soil microbial communities

Several effects of the catenas' different geochemistry and particle transport dynamics on the soil microbiology were observed in this study. Both geochemical and microbiological results pointed to a higher differentiation of soils along the granitic catena, while the basaltic soil samples were more similar to each other. This difference in structural and spatial differentiation between the two catenas is likely related to their different parent materials and topography. The increase in finer soil particles and nutrients along the granitic catena is in accordance with previous reports of soil nutrient content variation along hillslopes (e.g., Rogers and Tate, 2001; Venter et al., 2003; Khalili-Rad et al., 2011; Khomo et al., 2011) and likely due to particle transport dynamics, i.e., erosion, which are more pronounced in the sandy granitic soils compared to the basaltic soils. Effects of these nutrient dynamics on the soil microbiology of these catenas were reflected by positive correlations of (heterotrophic) activities, abundances of total *archaea* and of AOA in particular, and of bacterial community composition with soil nutrient contents. Additionally, the bacterial and archaeal phylum-level diversity was highest at the sampling locations GI and GII, which might be related to potentially higher spatial isolation and niche heterogeneity in these nutrient-limited upslope soils (Torsvik and Øvreås, 2002; Zhou et al., 2002). Similarly, a high small-scale heterogeneity and patchy distribution of archaeal soil communities has also been described for other soils (Timonen and Bomberg, 2009).

The sampling location above the seep zone (GII) exhibited a particularly distinct geochemistry and microbiology - likely because soils were subjected to seasonally fluctuating redox conditions above the actual seep zone, especially at sampling time near the end of the local wet season. Soils from GII were the most acidic and nutrient poor, showed high ammonium concentrations and the lowest microbial carbon use efficiency. The CCA performed with environmental parameters and bacterial 16S rRNA gene-based OTU frequencies also showed the strong distinction of this location from all other sampling sites. However, the variation underlying this separation could not fully be accounted for by the five tested environmental variables, indicating that variation here might be driven by potentially other relevant environmental parameters, such as redox potential or temporal fluctuations in water saturation, which were not assessed in our study. At this “above seep” location, several distinct bacterial and archaeal groups were also detected in higher relative abundances than in soils from the other sampling locations. *Ktedonobacteria*, for example, might have been abundant at GII due to the lower pH since the only cultured representative *K. racemifer* was also shown to be moderately acidophilic and to grow microaerophilically (Cavaletti et al., 2006). In the archaeal community, the RC-V was most abundant at GII, which agrees with their typical presence in temporarily anoxic habitats, e.g., rice fields (Großkopf et al., 1998; Ramakrishnan et al., 2000; Høj et al., 2008; Fu et al., 2015). Similarly, methanogenic *archaea* as detected at GII have previously been shown to occur in various soils (e.g., Australian savannas, tropical pastures, dry riverbeds) and to become active on short time scales under temporary wet, anoxic conditions (Angel et al., 2012; Scavino et al., 2013) as they might seasonally occur at GII due to fluctuating spatial extension of the active seep zone below.

## Conclusions

Our results have clearly demonstrated that the nutrient-poor conditions in South African savanna soils are a strong driver of microbial community structure, resulting in a dominance of *Actinobacteria, Chloroflexi*, and *Firmicutes* in the soil bacterial communities, which differs strongly from more nutrient-rich, temperate grassland soils. The oligotrophic status of the investigated soils was further reflected by an exceptionally high fraction of *archaea* in the soil microbial communities, which were mostly dominated by ammonia-oxidizing thaumarchaeota closely related to *Nitrososphaera* sp. The observed high numerical predominance of ammonia-oxidizing *archaea* compared to ammonia-oxidizing bacteria pointed to an important role of *archaea* in savanna soil nitrogen cycling, most likely in combination with nitrite oxidation by *Nitrospira*-related NOB. The general effect of the special properties of the savanna soils on the soil microbial communities was further modulated by the influence of bedrock material, resulting in the occurrence of some ammonia and nitrite oxidizers restricted to soils of either the granitic or basaltic catena.

Along the granitic catena, specific geochemical conditions and particle transport dynamics were found to affect the soil microbial communities through clay and nutrient relocation along the hill slope, probably underlying a shift to different, less diverse bacterial and archaeal communities at the downslope locations. Additionally, seasonal heterogeneity in soil water regime along the granitic catena may have supported the local occurrence of methanogenic *archaea* and Rice Cluster V *archaea* above a seasonal seep zone. Overall, our results suggest a strong effect of the savanna soils' nutrient scarcity and seasonal water limitations on all investigated microbial communities, resulting in the presence of distinct bacterial communities which differ strongly from those found in temperate grassland soils.

## Author contributions

KK, ST, and MH designed the work. ST, SL, and KK carried out field sampling. ST and SL contributed substantially to the biogeochemical, transect-oriented interpretation of the results. SR performed all molecular work and wrote the first version of the manuscript. Analysis of sequence data was carried out by SR and MH. SC contributed data of microbial respiration and biomass. CL performed the analysis of archaeal sequence data sets. All authors contributed to the writing of the manuscript.

### Conflict of interest statement

The authors declare that the research was conducted in the absence of any commercial or financial relationships that could be construed as a potential conflict of interest.
