# Corrigendum: Community Composition and Abundance of Bacterial, Archaeal, and Nitrifying Populations in Savanna Soils on Contrasting Bedrock Material in Kruger National Park, South Africa

**DOI:** 10.3389/fmicb.2016.01954

**Published:** 2016-11-29

**Authors:** Saskia Rughöft, Martina Herrmann, Cassandre S. Lazar, Simone Cesarz, Shaun R. Levick, Susan E. Trumbore, Kirsten Küsel

**Affiliations:** ^1^Chair of Aquatic Geomicrobiology, Institute of Ecology, Friedrich Schiller University JenaJena, Germany; ^2^German Centre for Integrative Biodiversity Research (iDiv) Halle-Jena-LeipzigLeipzig, Germany; ^3^Institute of Biology, Leipzig UniversityLeipzig, Germany; ^4^Biogeochemical Processes, Max Planck Institute for BiogeochemistryJena, Germany

**Keywords:** savanna soils, ammonia-oxidizing bacteria, ammonia-oxidizing archaea, amoA, granitic bedrock, basaltic bedrock

In the original article, we neglected to acknowledge the CRC 1076 “AquaDiva” funded by the Deutsche Forschungsgemeinschaft (DFG), who provided financial support for this project. The authors apologize for this oversight. This error does not change the scientific conclusions of the article in any way.

## Funding

MiSeq Illumina amplicon sequencing was financially supported by the German Center for Integrative Biodiversity Research (iDiv) Halle-Jena-Leipzig, funded by the German Research Foundation (FZT 118). ST acknowledges the AW Mellon foundation for their support through grant #41200677. This work was financially supported by the CRC 1076 AquaDiva, funded by the German Research Foundation.

### Conflict of interest statement

The authors declare that the research was conducted in the absence of any commercial or financial relationships that could be construed as a potential conflict of interest.

