# Supplementary material for: Community Composition and Abundance of Bacterial, Archaeal and Nitrifying Populations in Savanna Soils on Contrasting Bedrock Material in Kruger National Park, South Africa
Source: Front Microbiol. 2016 Oct 19;7:1638. doi: 10.3389/fmicb.2016.01638 (PMC5069293; doi:10.3389/fmicb.2016.01638)
Supplement: Supplementary file 1 [file Table1.PDF]

Supplementary Table 1. Primer sequences. Primers used for the amplification of bacterial and archaeal 16S rRNA genes, archaeal and bacterial *amoA* genes and *nxrB* genes used in this study.

application	targeted gene	Primer	5'-3' sequence	Reference
clone library construction, qpCR	archaeal *amoA*	Arch-AmoAF	STAATGGTCTGGCTTAGACG	Francis *et al.* 2005
		Arch-AmoAR	GCGGCCATCCATCTGTATGT	
clone library construction, qPCR	bacterial *amoA*	AmoA-1F	GGGGTTTCTACTGGTGGT	Rotthauwe *et al.* 1997
		AmoA-2R	CCCCTCKGSAAAGCCTTCTTC	
clone library construction	*nxrB* of *Nitrospira*	nxrB19	TGGCAACTGGGACGGAAGATG	Pester *et al.* 2014
		nxrB1237r	GTAGATCGGCTCTTCGACCTG	
Illumina MiSeq sequencing	bacterial 16S rRNA	Bakt_314F	CCTACGGGNGGCWGCAG	Herlemann *et al.* 2011
		Bakt_805R	GACTACHVGGGTATCTAATCC	
Illumina MiSeq sequencing	archaeal 16S rRNA	Parch349F	CCCTACGGGGYGCASCAG	Ovreås *et al. 1997*
		Arch915r	GTGCTCCCCCGCCAATTCCT	Stahl *et al.* 1991
qPCR	bacterial 16S rRNA	Bac8Fmod	AGAGTTTGATYMTGGCTCAG	Daims *et al.* 1999
		Bac338Rabc	GCWGCCWCCCGTAGGWGT	Loy *et al.* 2002
qPCR	archaeal 16S rRNA	Arch806F	ATTAGATACCCSBGTAGTCC	Takai & Horikoshi 2000
		Arch958R	YCCGGGGTTGAMTCCAATT	Loy *et al.* 2002
re-amplification for DGGE	archaeal *amoA*	Arch-AmoAF(short)	TAATGGTCTGGCTT	Herrmann *et al.* 2011
		Arch-AmoAR-GC	CGC CCG CCG CGC GGC GGG CGG GGC GGG GGC ACG GGG-GCG GCC ATC CAT CTG TAT GT	
