# Supplementary material for: Community Composition and Abundance of Bacterial, Archaeal and Nitrifying Populations in Savanna Soils on Contrasting Bedrock Material in Kruger National Park, South Africa
Source: Front Microbiol. 2016 Oct 19;7:1638. doi: 10.3389/fmicb.2016.01638 (PMC5069293; doi:10.3389/fmicb.2016.01638)
Supplement: Supplementary file 2 [file Table2.PDF]

Supplementary Table 2. Illumina sequencing results targeting bacterial and archaeal 16S rRNA genes after normalization (subsampling) and singleton removal. The number of respective reads after sequence analysis, numbers of observed OTUs, estimated OTU numbers (Chao1 richness estimator), the sequencing coverage (SC) and the Shannon diversity index (SDI) for each targeted gene are shown per soil sample (triplicates = a/b/c). For some samples, selected replicates were excluded from analysis due to read numbers being too low.

Sample code	Bacterial 16S rRNA genes					Archaeal 16S rRNA genes				
	Reads	OTUs	Chao1	SC	SDI	reads	OTUs	Chao1	SC	SDI
**GI_a**	3812	934	1489	0.88	5.83	1192	42	56	0.99	2.02
**GI_b**	4030	834	1302	0.90	5.14	1189	38	38	1.00	2.15
**GI_c**	4191	982	1409	0.90	5.94	1183	39	43	0.99	1.49
**GII_a**	4140	887	1194	0.91	5.73	1189	44	63	0.99	3.12
**GII_b**	4066	979	1498	0.89	5.87	-	-	-	-	-
**GII_c**	-	-	-	-	-	1208	51	58	0.99	2.87
**GIII_a**	4120	695	1124	0.92	5.14	1188	23	41	0.99	2.01
**GIII_b**	4027	654	995	0.92	5.19	1199	18	27	0.99	0.93
**GIII_c**	-	-	-	-	-	1201	12	15	1.00	1.17
**GIV_a**	3819	782	1297	0.89	4.51	1185	51	62	0.99	2.16
**GIV_b**	-	-	-	-	-	-	-	-	-	-
**GIV_c**	-	-	-	-	-	1189	44	51	0.99	1.74

Supplementary Table 2, continued

Sample code	Bacterial 16S rRNA genes					Archaeal 16S rRNA genes				
	Reads	OTUs	Chao1	SC	SDI	reads	OTUs	Chao1	SC	SDI
**BI_a**	-	-	-	-	-	1191	33	36	1.00	2.11
**BI_b**	4244	688	1087	0.92	5.13	-	-	-	-	-
**BI_c**	4327	660	893	0.94	5.06	-	-	-	-	-
**BII_a**	3906	822	1446	0.89	5.29	1183	30	39	0.99	1.82
**BII_b**	3827	738	1274	0.89	4.78	1186	29	62	0.99	1.76
**BII_c**	3786	728	1382	0.89	4.37	1176	31	91	0.99	1.71
**BIII_a**	4137	698	1293	0.91	4.79	1201	34	36	1.00	2.09
**BIII_b**	4284	634	1183	0.93	4.76	1196	29	35	0.99	1.69
**BIII_c**	-	-	-	-	-	-	-	-	-	-
