# Supplementary material for: Community Composition and Abundance of Bacterial, Archaeal and Nitrifying Populations in Savanna Soils on Contrasting Bedrock Material in Kruger National Park, South Africa
Source: Front Microbiol. 2016 Oct 19;7:1638. doi: 10.3389/fmicb.2016.01638 (PMC5069293; doi:10.3389/fmicb.2016.01638)
Supplement: Supplementary file 3 [file Table3.PDF]

Supplementary Table 3. Results of cloning and sequencing of bacterial and archaeal *amoA* genes and *nxrB* genes. Distance cutoffs used for OTU clustering: 0.20 for the bacterial *amoA* gene, 0.15 for the archaeal *amoA* gene, 0.05 for the *nxrB* gene.

clone library	sample	number of clones	observed OTUs
bacterial *amoA* gene	GI	25	1
	GIV	20	
	BII	30	
archaeal *amoA* gene	GI	21	12
	GIV	28	
	BII	30	
nxrB gene	GI	30	16
	BII	35	
