# Supplementary material for: Community Composition and Abundance of Bacterial, Archaeal and Nitrifying Populations in Savanna Soils on Contrasting Bedrock Material in Kruger National Park, South Africa
Source: Front Microbiol. 2016 Oct 19;7:1638. doi: 10.3389/fmicb.2016.01638 (PMC5069293; doi:10.3389/fmicb.2016.01638)
Supplement: Supplementary file 4 [file Table4.PDF]

Supplementary Table 4. Illumina sequencing results targeting bacterial and archaeal 16S rRNA genes before normalization and singleton removal. The number of respective reads after sequence analysis, numbers of observed OTUs, estimated OTU numbers (Chao1 richness estimator), the sequencing coverage (SC) and the Shannon diversity index (SDI) for each targeted gene are shown per soil sample (triplicates = a/b/c).

Sample code	Bacterial 16S rRNA genes					Archaeal 16S rRNA genes				
	Reads	OTUs	Chao1	SC	SDI	reads	OTUs	Chao1	SC	SDI
**GI_a**	10053	2774	8065	0.81	6.54	2624	148	1347	0.96	2.31
**GI_b**	10798	2311	5799	0.87	5.79	2163	127	1106	0.96	2.42
**GI_c**	4516	1307	2655	0.84	6.19	6429	316	1885	0.96	1.89
**GII_a**	7819	1735	3310	0.88	6.15	1755	137	381	0.96	3.34
**GII_b**	4518	1429	3441	0.80	6.22	499	52	239	0.93	2.29
**GII_c**	1915	709	1833	0.75	5.57	1241	84	256	0.97	3.01
**GIII_a**	5980	1300	3602	0.86	5.57	4228	211	2061	0.96	2.32
**GIII_b**	8560	1799	5262	0.86	5.78	9057	373	5204	0.96	1.32
**GIII_c**	3011	893	2774	0.79	5.63	11683	382	4770	0.97	1.49
**GIV_a**	10143	2753	9393	0.80	5.49	15190	680	4551	0.96	2.52
**GIV_b**	76	51	154	0.46	3.64	22	13	36	0.55	2.26
**GIV_c**	1956	860	2270	0.69	5.85	2535	164	648	0.95	2.05

Supplementary Table 4, continued

Sample code	Bacterial 16S rRNA genes					Archaeal 16S rRNA genes				
	Reads	OTUs	Chao1	SC	SDI	reads	OTUs	Chao1	SC	SDI
**BI_a**	3256	889	2417	0.81	5.47	1308	85	85	0.96	2.36
**BI_b**	8186	1404	3337	0.89	5.49	195	36	36	0.92	2.55
**BI_c**	9474	1340	2698	0.92	5.35	439	43	43	0.96	2.37
**BII_a**	12185	2886	9750	0.83	6.04	13032	698	698	0.95	2.24
**BII_b**	9928	2652	10657	0.80	5.71	6190	318	318	0.96	2.16
**BII_c**	14190	3393	12732	0.82	5.42	18050	713	713	0.96	2.07
**BIII_a**	7277	1523	4304	0.86	5.28	1698	93	93	0.96	2.31
**BIII_b**	8460	1300	3910	0.90	5.08	6630	307	307	0.96	2.04
**BIII_c**	-	-	-	-	-	61	58	58	0.10	4.04
