# Supplementary material for: Community Composition and Abundance of Bacterial, Archaeal and Nitrifying Populations in Savanna Soils on Contrasting Bedrock Material in Kruger National Park, South Africa
Source: Front Microbiol. 2016 Oct 19;7:1638. doi: 10.3389/fmicb.2016.01638 (PMC5069293; doi:10.3389/fmicb.2016.01638)
Supplement: Supplementary file 5 [file Presentation1.PDF]

Supplementary Material

Nutrient poor South African savanna soils on contrasting bedrock material harbor distinct microbial communities dominated by Actinobacteria, Chloroflexi, Firmicutes and ammonia oxidizing archaea

Saskia Rughöft, Martina Herrmann, Cassandre S. Lazar, Simone Cesarz, Shaun R. Levick, Susan Trumbore, Kirsten Küsel*

*** Correspondence:** Kirsten.Kuesel@uni-jena.de

1 Supplementary Methods

1.1 DGGE analysis of archaeal *amoA* genes

PCR products for DGGE analysis of archaeal *amoA* genes were generated in a semi-nested PCR approach. Purified archaeal *amoA* PCR products obtained with primer set Arch-AmoAF/Arch-AmoAR (Francis *et al.* 2005) were used as templates in a second PCR with primers Arch-AmoAF(short)/Arch-AmoAR-GC (Herrmann *et al.* 2011) and with the following cycling conditions: 95°C for 10 min; 19 cycles of 94°C for 60 sec, 53°C for 45 sec and 72°C for 60 sec; and 72°C for 10 min. Re-amplification was performed in a total volume of 50 µl containing 5.0 µl of 10x High Yield Buffer complete and 1.5 U *Taq* polymerase (Jena Bioscience, Germany), 0.2 µM of each primer, 0.08 mM of dNTP Mix (Thermo Fisher Scientific) and 2.0 µl of purified PCR product. PCR products were separated on 8% polyacrylamide gels with a denaturant gradient of 30% to 60% (as defined by Muyzer *et al.* 1997) at 100 V and 60°C for 16 h (INGENYphorU-2 DGGE system, Ingeny, The Netherlands). Gels were stained with SYBR Gold (Invitrogen) and DGGE band patterns were used to construct presence/absence matrices for each sample which were then subjected to a cluster analysis (algorithm: paired groups; similarity measure: dice; Bootstrapping: N = 100) using the PAST software package (V3.07) by Hammer *et al.* (2001). DGGE was only performed with archaeal *amoA* amplicons due to too low abundances of bacterial *amoA* genes in the samples.

References

Daims, H., Brühl, A., Amann, R., Schleifer, K.-H., Wagner, M. (1999). The domain-specific probe EUB338 is insufficient for the detection of all bacteria: development and evaluation of a more comprehensive probe set. *Systematic and Applied Microbiology* 22: 434–444.

Francis, C.A., Roberts, K.J., Beman, J.M., Santoro, A.E., Oakley, B.B. (2005). Ubiquity and diversity of ammonia-oxidizing archaea in water columns and sediments of the ocean. *Proceedings of the National Academy of Sciences of the United States of America* 102, 14683–14688.

Hammer, Ø., Harper, D.A.T., Ryan, P.D. (2001). PAST: Paleontological statistics software package for education and data analysis. *Palaeontologia Electronica* 4, 1.

Herlemann, D.P.R., Labrenz, M., Juergens, K., Bertilsson, S., Waniek, J.J., Anderrson. A.F. (2011). Transition in bacterial communities along the 2000 km salinity gradient of the Baltic Sea. *The ISME Journal* 5, 1571-1579.

Herrmann, M., Scheibe, A., Avrahami, S., Küsel, K. (2011). Ammonium Availability Affects the Ratio of Ammonia-Oxidizing Bacteria to Ammonia-Oxidizing Archaea in Simulated Creek Ecosystems. *Applied and Environmental Microbiology* 77, 5, 1896-1899.

Loy, A., Lehner, A., Lee, N., Adamczyk, J., Meier, H., Ernst, J., et al. (2002). Oligonucleotide Microarray for 16S rRNA Gene-Based Detection of All Recognized Lineages of Sulfate-Reducing Prokaryotes in the Environment. *Applied and Environmental Microbiology* 68, 10, 5064-5081.

Muyzer, G., Brinkhoff, T., Nübel, U., Santegoeds, C., Schafer, H., Wawer, C. (1997). Denaturing gradient gel electrophoresis (DGGE). in microbial ecology. In Akkermans A.D.L., van Elsas J.D., Bruijn J.F. (Eds.)., *Molecular Microbial Ecology Manual*, 1-27. Kluwer Academic Publishers, Dordrecht, The Netherlands.

Øvreås, L., Forney, L., Daae, F.L., Torsvik, V. (1997). Distribution of bacterioplankton in meromictic Lake Saelenvannet, as determined by denaturing gradient gel electrophoresis of PCR-amplified gene fragments coding for 16S rRNA. *Applied Environmental Microbiology* 63, 3367-3373.

Pester, M., Maixner, F., Berry, D., Rattel, T., Koch, H., Lücker, S., et al. (2014). NxrB encoding the beta subunit of nitrite oxidoreductase as functional and phylogenetic marker for nitrite-oxidizing Nitrospira. *Environmental Microbiology* 16, 10, 3055-3071.

Rotthauwe, J.-H., Witzel, K.-P., Liesack, W. (1997). The Ammonia Monooxygenase Structural Gene amoA as a Functional Marker: Molecular Fine-Scale Analysis of Natural Ammonia-Oxidizing Populations. *Applied and Environmental Microbiology* 63, 12, 4704–4712.

Stahl, D.A., Amann, R. (1991). Development and application of nucleic acid probes. In E. Stackebrandt and M. Goodfellow (Ed.)., *Nucleic acid techniques in bacterial systematics*, 205-248. John Wiley & Sons, Chichester, England.

Takai, K. & Horikoshi, K. (2000). Rapid Detection and Quantification of Members of the Archaeal Community by Quantitative PCR Using Fluorogenic Probes. *Applied and Environmental Microbiology* 66, 11, 5066-5072.
