# Supplementary material for: Community Composition and Abundance of Bacterial, Archaeal and Nitrifying Populations in Savanna Soils on Contrasting Bedrock Material in Kruger National Park, South Africa
Source: Front Microbiol. 2016 Oct 19;7:1638. doi: 10.3389/fmicb.2016.01638 (PMC5069293; doi:10.3389/fmicb.2016.01638)
Supplement: Supplementary file 7 [file Image2.pdf]

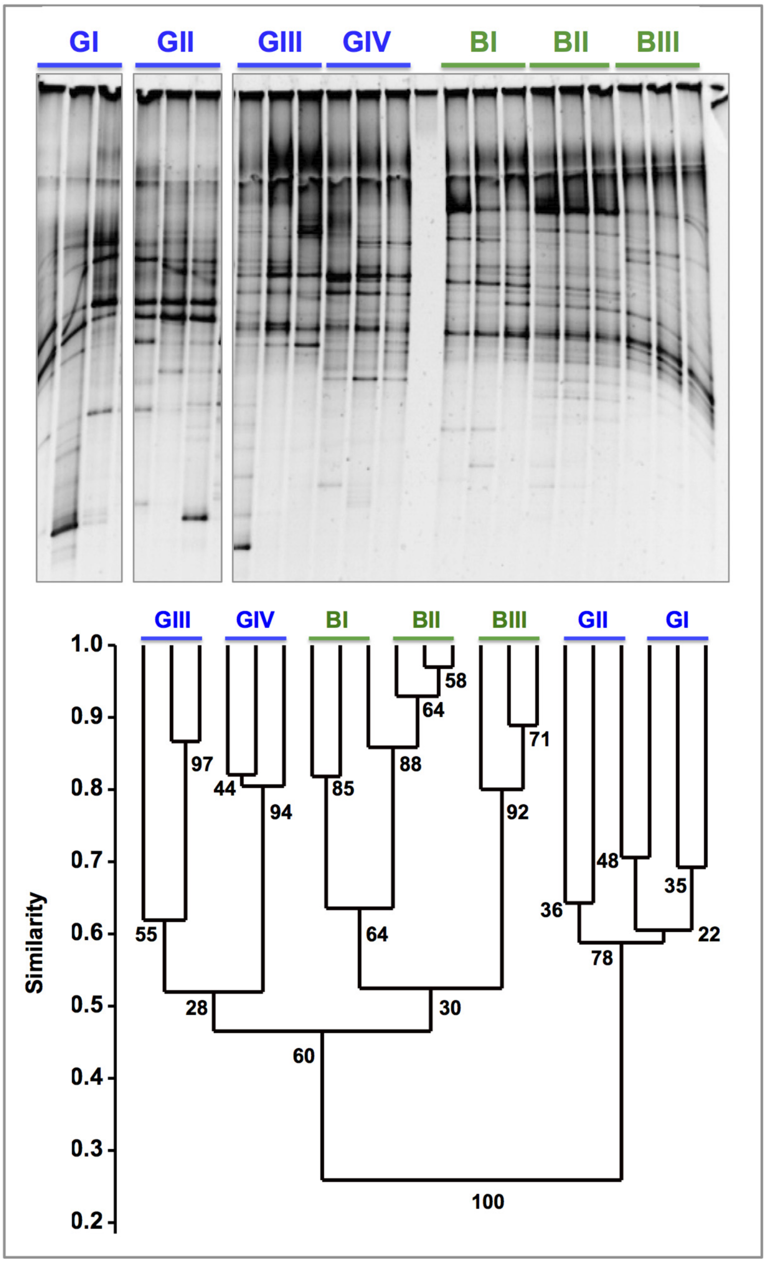

Supplementary Figure 2. Band patterns from archaeal *amoA* DGGE. Top: DGGE fingerprint in a 30% to 60% denaturing gradient gel of the archaeal *amoA* genes obtaimed from the soil samples taken along the granitic (GI-GIV) and basaltic (BI-BIII) catena. Bottom: Cluster analysis of band patterns (algorithm: paired group; similarity measure: dice; Boot N = 100) of the DGGE gel pictured above.
