# Supplementary material for: Community Composition and Abundance of Bacterial, Archaeal and Nitrifying Populations in Savanna Soils on Contrasting Bedrock Material in Kruger National Park, South Africa
Source: Front Microbiol. 2016 Oct 19;7:1638. doi: 10.3389/fmicb.2016.01638 (PMC5069293; doi:10.3389/fmicb.2016.01638)
Supplement: Supplementary file 8 [file Image3.PDF]

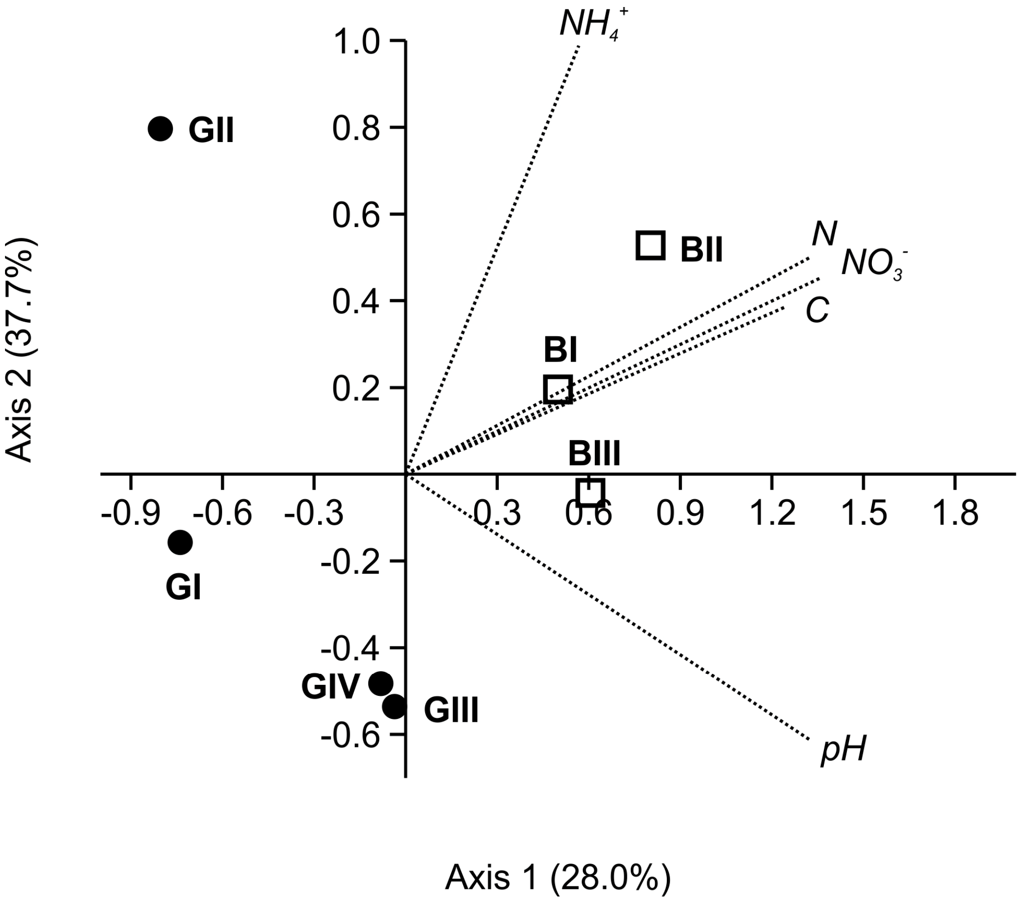

Supplementary Figure 3. Biplot from canonical correspondence analysis (CCA) of six measured environmental variables (ammonium concentration, nitrate concentration, total C, total N, soil pH) and the archaeal *amoA*-based community structure in soil samples from two catenas, detected as DGGE band patterns of sampling locations. Samples from the granitic and the basaltic catena are shown as filled circles (GI-GIV) and open squares (BI-BIII), respectively.
