# Supplementary material for: Community Composition and Abundance of Bacterial, Archaeal and Nitrifying Populations in Savanna Soils on Contrasting Bedrock Material in Kruger National Park, South Africa
Source: Front Microbiol. 2016 Oct 19;7:1638. doi: 10.3389/fmicb.2016.01638 (PMC5069293; doi:10.3389/fmicb.2016.01638)
Supplement: Supplementary file 9 [file Image4.PDF]

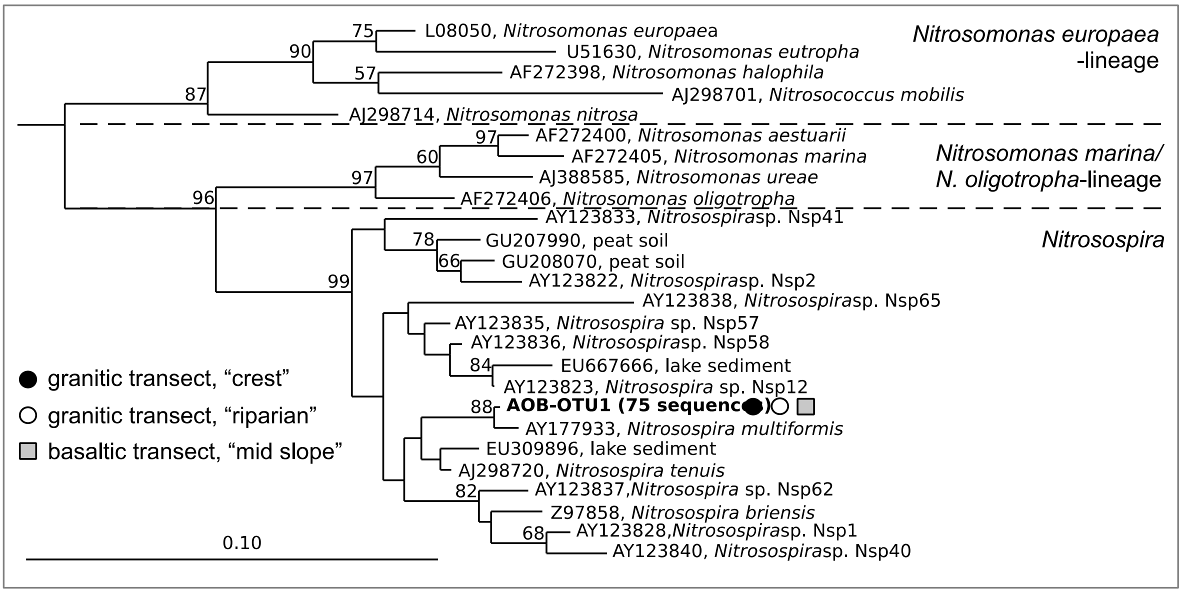

Supplementary Figure 4: Phylogenetic tree of deduced bacterial AmoA protein sequences. For all observed OTUs from the constructed bacterial *amoA* clone libraries, one representative sequence is shown and the total number of clones per OTU is given in parentheses. The tree was constructed via neighbour-joining (Boot N = 1000). The origin of all sequences assigned to each OTU regarding the sampling sites (granitic and basaltic catena; GI, GIV, BII) is indicated by filled or open circles or squares, respectively. Only bootstrap values higher than 50% are shown.
