# Supplementary material for: Community Composition and Abundance of Bacterial, Archaeal and Nitrifying Populations in Savanna Soils on Contrasting Bedrock Material in Kruger National Park, South Africa
Source: Front Microbiol. 2016 Oct 19;7:1638. doi: 10.3389/fmicb.2016.01638 (PMC5069293; doi:10.3389/fmicb.2016.01638)
Supplement: Supplementary file 11 [file Image6.PDF]

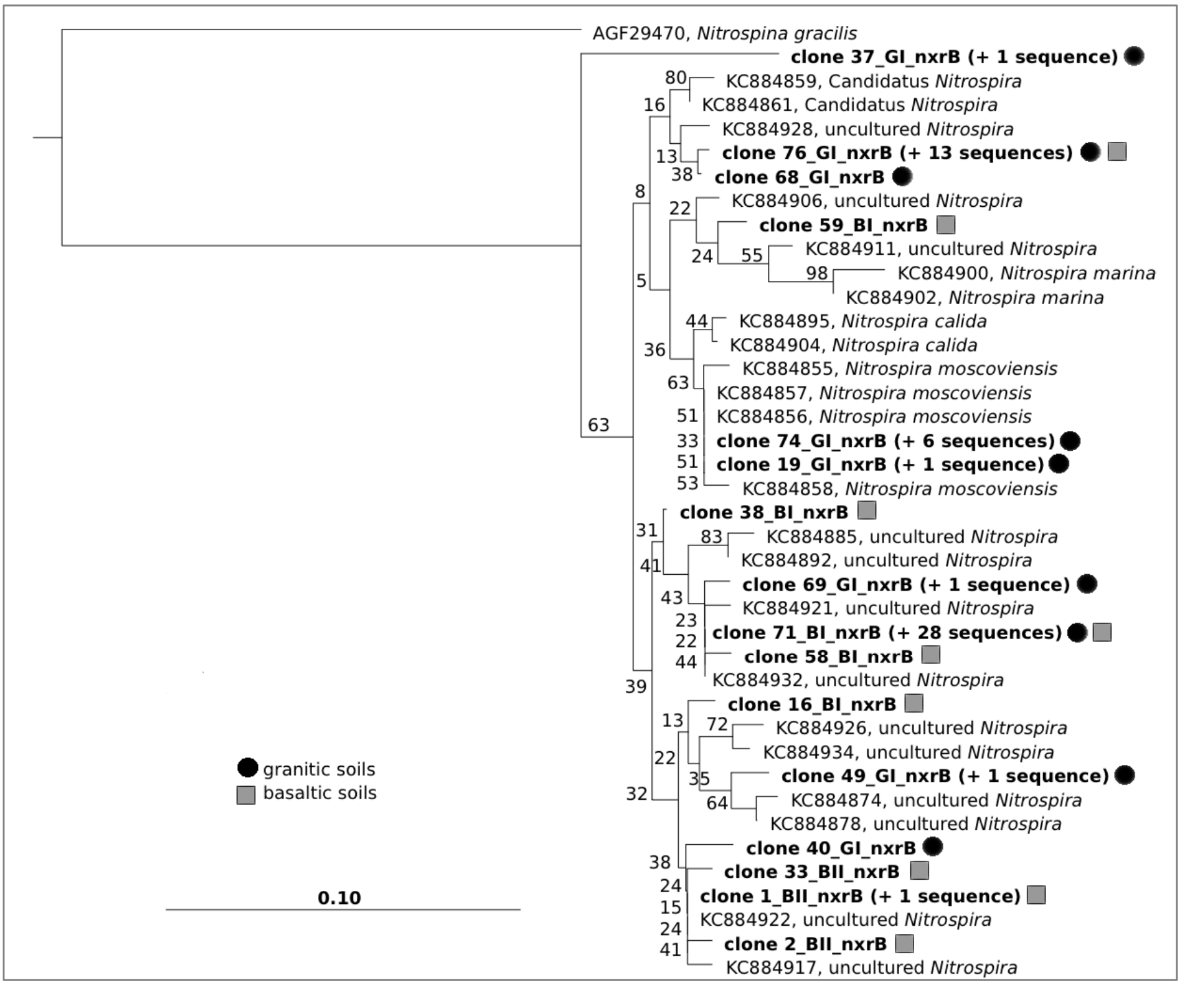

Supplementary Figure 6: Phylogenetic tree of deduced NxrB protein sequences. For all observed OTUs from the constructed *nxrB* clone libraries, one representative sequence is shown and the total number of clones per OTU is given in parentheses. The tree was constructed via neighbour-joining (Boot N = 1000). The origin of all sequences assigned to each OTU regarding the sampling sites (granitic and basaltic catena; GI and BII) is indicated by filled circles or squares, respectively.
