# Supplementary material for: Community Composition and Abundance of Bacterial, Archaeal and Nitrifying Populations in Savanna Soils on Contrasting Bedrock Material in Kruger National Park, South Africa
Source: Front Microbiol. 2016 Oct 19;7:1638. doi: 10.3389/fmicb.2016.01638 (PMC5069293; doi:10.3389/fmicb.2016.01638)
Supplement: Supplementary file 12 [file Image7.PDF]

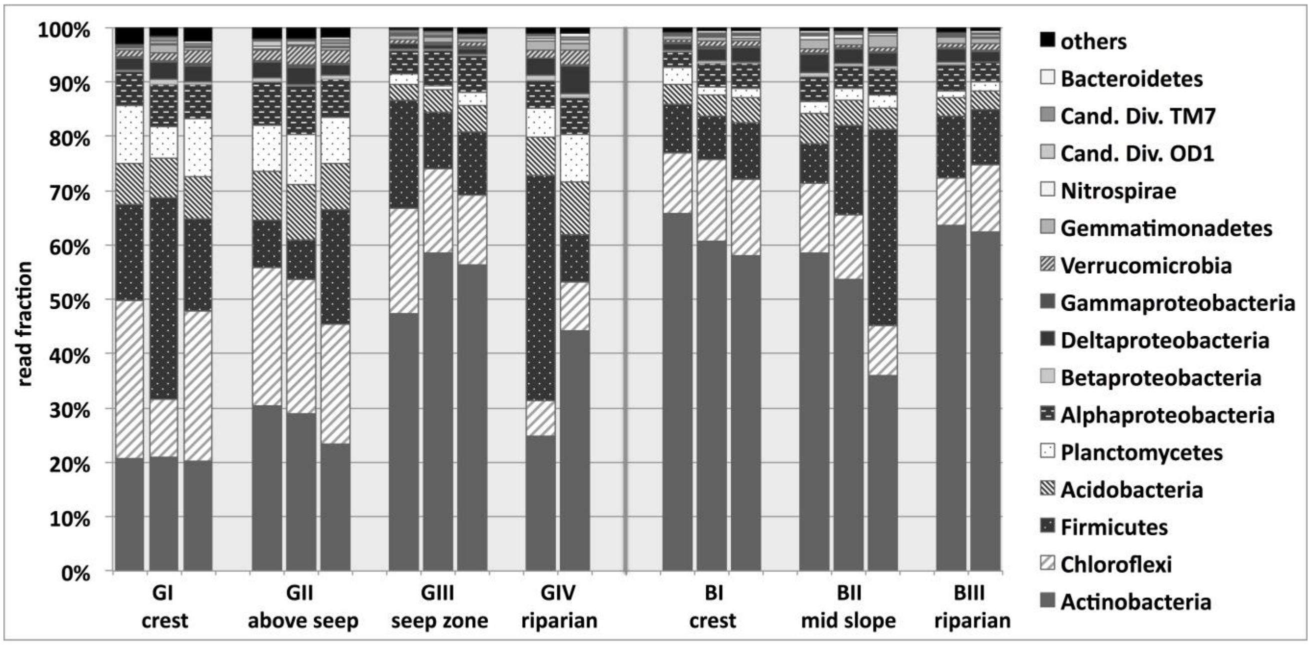

Supplementary Figure 7: Phlyum-level taxonomic classification of sequences obtained from bacterial 16S rRNA gene-targeted Illumina sequencing of savanna soil samples taken from 5 cm depth at different locations along the granitic (GI-GIV) and the basaltic (BI-BIII) catena. Cand. Div. = Candidate Division. Results are based on sequence data before normalization and removal of singletons.
