# Supplementary material for: Community Composition and Abundance of Bacterial, Archaeal and Nitrifying Populations in Savanna Soils on Contrasting Bedrock Material in Kruger National Park, South Africa
Source: Front Microbiol. 2016 Oct 19;7:1638. doi: 10.3389/fmicb.2016.01638 (PMC5069293; doi:10.3389/fmicb.2016.01638)
Supplement: Supplementary file 13 [file Image8.PDF]

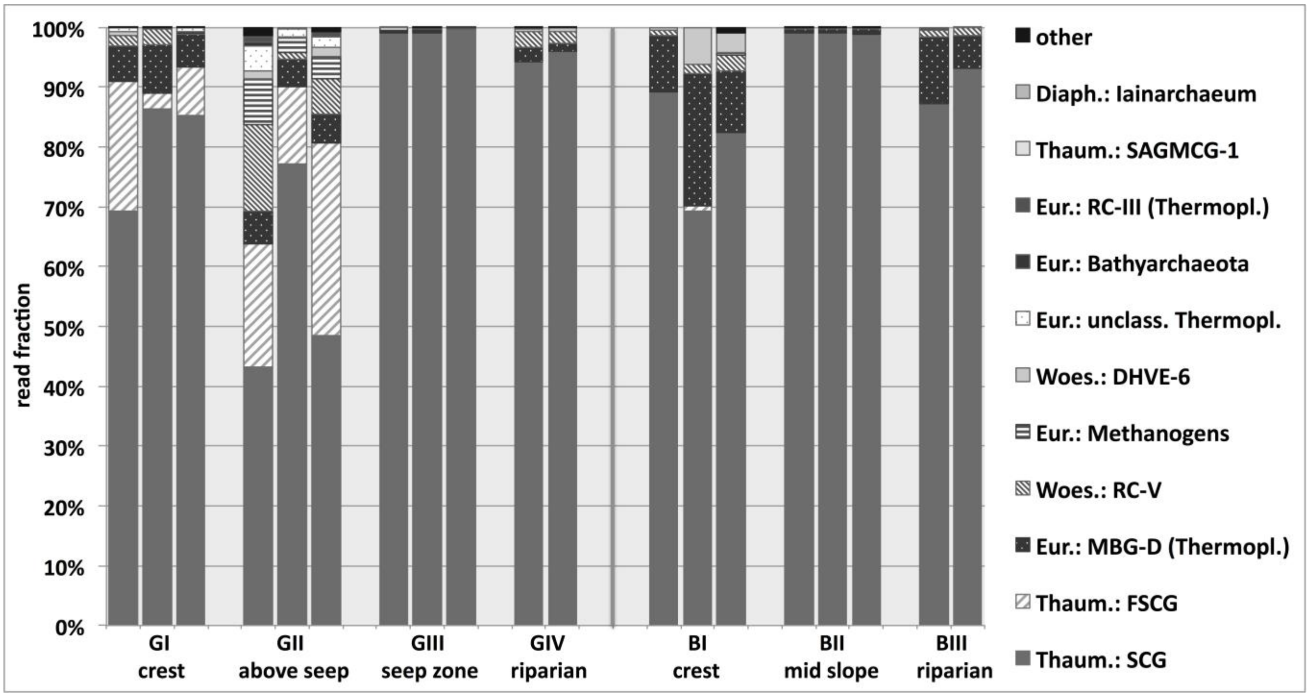

Supplementary Figure 8: : Classification of sequences obtained from archaeal 16S rRNA gene-targeted Illumina sequencing of savanna soil samples taken from 5 cm depth at different locations along the granitic (GI-GIV) and the basaltic (BI-BIII) catena. Diaph. = Diapherotrites; Thaum. = Thaumarchaeota; SAGMG = South African Gold Mine Group; Eur. = Euryarchaeota; RC = Rice Cluster; Thermopl. = Thermoplasmatales; unclass. = unclassified; Woes. = Woesearchaeota; DHVE = Deep Sea Hydrothermal Vent Euryarchaeota Group; MBG = Marine Benthic Group; (F)SCG = (Forest) Soil Creanarchaeota Group. Results are based on sequence data before normalization and removal of singletons.
